# The ephemeral fumarolic mineralization of the 2021 Tajogaite volcanic eruption (La Palma, Canary Islands, Spain)

**DOI:** 10.1038/s41598-023-33387-6

**Published:** 2023-04-18

**Authors:** Marc Campeny, Inmaculada Menéndez, Jordi Ibáñez-Insa, Jesús Rivera-Martínez, Jorge Yepes, Soledad Álvarez-Pousa, Jorge Méndez-Ramos, José Mangas

**Affiliations:** 1 Departament de Mineralogia, Museu de Ciències Naturals de Barcelona, Passeig Picasso s/n, 08003 Barcelona, Spain; 2 Instituto de Oceanografía y Cambio Global, IOCAG, Dept. de Física, Universidad de Las Palmas de Gran Canaria, 35017 Las Palmas de Gran Canaria, Spain; 3 Geosciences Barcelona (GEO3BCN), Spanish Council for Scientific Research (CSIC), Lluís Solé i Sabarís s/n, 08028 Barcelona, Spain; 4 Agencia Estatal de Investigación, Calle Torrelaguna 58, 28027 Madrid, Spain; 5 Instituto de Materiales y Nanotecnología, Departamento de Física, Universidad de La Laguna, apdo correos 456, 38200 La Laguna, Tenerife Spain; 6 Instituto de Oceanografía y Cambio Global, IOCAG, Dept. de Ingeniería Civil, Universidad de Las Palmas de Gran Canaria, 35017 Las Palmas de Gran Canaria, Spain

**Keywords:** Geology, Mineralogy, Volcanology

## Abstract

The present work aims to characterize the ephemeral mineral assemblage related to the fumarolic fields of the Tajogaite volcano, formed in 2021 in La Palma Island (Canary Islands, Spain). A set of 73 samples was obtained after two sampling campaigns in different fumarole sectors of the studied area. Mineralization related to these fumaroles formed efflorescent patches located at variable distance from the main volcanic craters. Distal patches are predominantly whitish, while in the vicinities they typically show yellowish to orange colours. Field observations also revealed that fumaroles usually occur in elevated topographic areas as well as over fractured and porous volcanic pyroclastic materials. The mineralogical and textural characterisation of the Tajogaite fumaroles unfolds a complex mineral assemblage, comprising cryptocrystalline phases related to low (< 200 °C) and medium temperature (200–400 °C) conditions. In Tajogaite, we propose a classification of three different fumarolic mineralization types: (1) fluorides and chlorides located in proximal fumarolic areas (~ 300–180 °C); (2) native sulphur associated with gypsum, mascagnite and salammoniac (~ 120–100 °C) and (3) sulphates and alkaline carbonates typically occurred in distal fumarolic areas (< 100 °C). Finally, we present a schematic model of the formation of Tajogaite fumarolic mineralization and their compositional evolution developed during the cooling of the volcanic system.

## Introduction

Volcanic eruptions are generally followed by different phenomena related to magma cooling. Fumaroles, superficial vents that emit hot gases associated with volcanic degassing, are among the most common post-eruption processes. They emit large volumes of steam but also variable amounts of other magmatic gases such as CO_2_, CO, SO_2_, H_2_S, HCl, HF, H_2_, NH_4_ or CH_4_ among others^1–3^. This variable composition of gases and their corresponding interaction with the volcanic rocks generate a highly peculiar and complex mineralization associated with fumarolic environments.

Minerals developed during fumarolic activity occur as cryptocrystalline or microcrystalline aggregates, typically mixed with other phases. This mineral assemblage is directly controlled by the compositional features of the magmatic system but also by other factors such as temperature. In fact, fumarole minerals can be classified in two separate groups: (1) sublimates, originated by gas phase condensation, and (2) incrustations, which are the product of interactions between fumarolic gases and wall rock^4^.

In recent years, there has been growing interest in the study of this type of mineralization due to the idea that fumaroles may be possible environments involved in the origin of life on Earth^5–7^. Studies on efflorescent minerals associated with fumaroles have also been relevant in planetary science, since this type of mineral assemblage has been recently reported in Mars^8–10^.

Unfortunately, despite their unquestionable interest, fumarolic minerals are very unstable and sensitive to weathering and, consequently, cannot be preserved in the geological register. The study and characterization of these ephemeral phases is therefore restricted to volcanic areas where recent eruptions have occurred or to exceptionally long-life volcanic sectors with a continuous and significant geothermal activity (e.g., Yellowstone in the USA or Taupo in New Zealand).

One of the most recent opportunities to investigate fumarole mineralization was provided by the recent Tajogaite eruption, which occurred during the last quarter of 2021 in La Palma Island, Canary Islands, Spain. This volcano quickly raised great interest among the scientific community, and many groups from different institutions have been intensively working to characterize numerous aspects of the eruption. In particular, Martinez-Martinez et al.^11^ have very recently reported a first characterization of Tajogaite’s fumarolic minerals, with special emphasis in the morphology of the minerals.

The aim of the present work is to characterize the mineralogy associated with the fumaroles of the Tajogaite volcano and to contribute to the general knowledge of these exotic types of mineralization and their relation to different factors of the volcanic system. In particular, here we focus our work on the fumarolic mineral paragenesis distribution as well as the genetical conditions and geomorphological control of this ephemeral mineralization.

## Geological setting

### La Palma regional geology

The Canary Islands archipelago is part of the Canary Islands Seamounts Province (CISP). This volcanic province is located on a passive continental margin which extends parallel to the NW African continental shelf^12^. The CISP is genetically related to a hotspot intraplate system and, according to measurements of magnetic anomalies and ages obtained in emerged rocks as well as in the submarine seamounts, shows evidence of volcanism from the Early Cretaceous^13^. Although records of Quaternary subaerial volcanism exist in all the Canary Islands except La Gomera, the largest volume of recently ejected material is found in La Palma and El Hierro, the westernmost islands of the archipelago^13^. These two islands date to the Plio-Quaternary (< 4 Ma)^14,15^.

**Figure 1:**
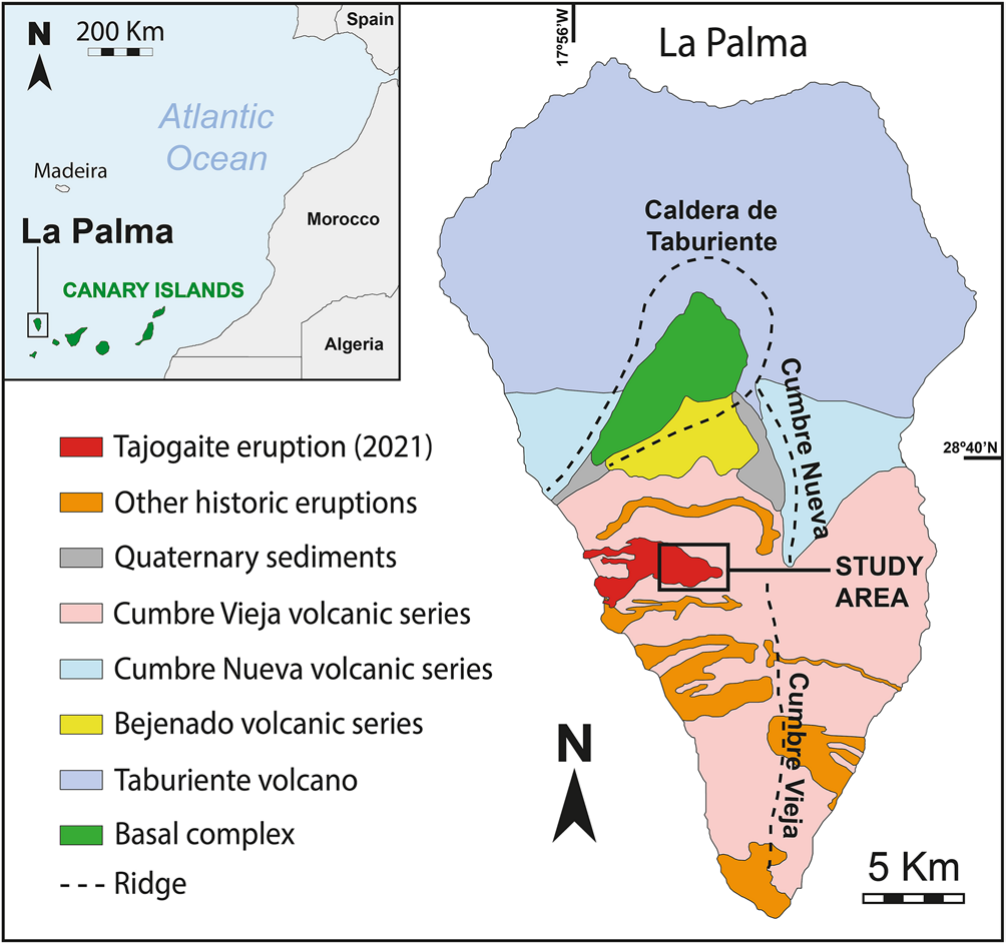
Geological map of La Palma. Location of the historical volcanic eruptions reported in the island, including the latest one which occurred in the Tajogaite area during 2021 that partially corresponds to the present work study area.

In the particular case of La Palma, volcanic activity began with the formation of the submarine complex (3–4 Ma)^16–18^. Subsequently, the island emerged with the development of the Garafía and the Taburiente shield volcanoes (1.7–0.4 Ma)^14^ as well as the youngest volcanic structures of the Cumbre Nueva rift (850–560 ka)^17^ and the Bejenado complex, which is located in the SW landslide scar of the Taburiente volcano (490–560 ka; Fig. 1)^14,17^. Finally, volcanism spread southwards forming the Cumbre Vieja rift (ca. 125 ka), which hosts the nowadays active volcanic system in La Palma (Fig. 1)^17,19–21^.

Considering historical times, La Palma is the most active volcanic area of the Canary Islands archipelago. Up to 8 eruptions have been documented since the fifteenth century: ~ 1480 (Montaña Quemada), 1585 (Tajuya), 1677 (San Antonio), 1646 (Martín), 1712 (El Charco), 1949 (San Juan)^22^, 1971 (Teneguía) and, finally, the Tajogaite eruption which occurred in 2021 (Fig. 1)^23–28^.

### The Tajogaite eruption

**Figure 2:**
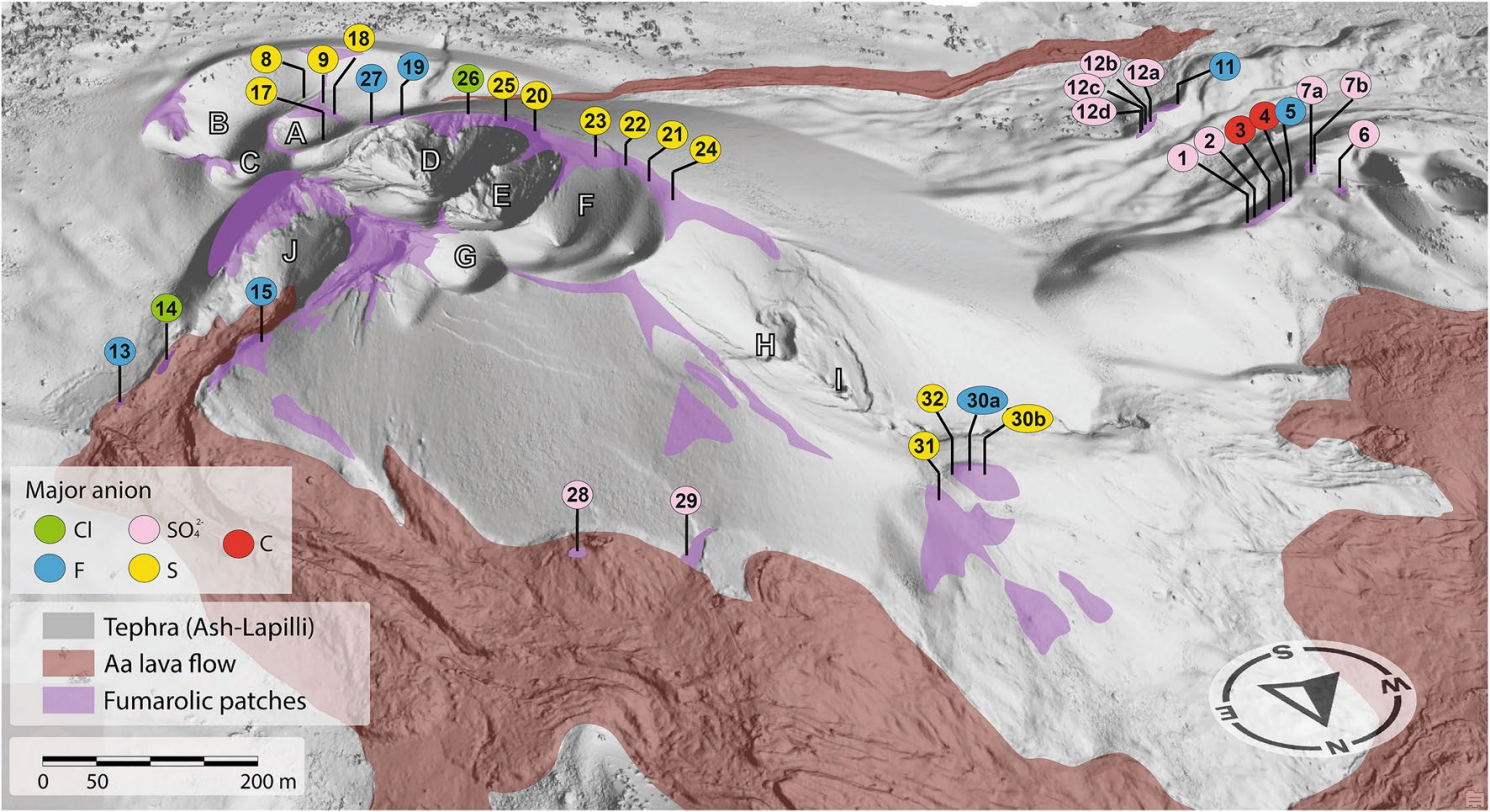
Northern 3D view of the Tajogaite volcano cone. Major mineralization patches related to fumarole activity are highlighted in purple. Individual craters are labelled by letters (A–J) and samples by numbers (1–32). The Digital Elevation Model has been modified from Cívico et al.^29^.

From 19 September and lasting until 13 December 2021 (85 days), after 50 years of volcanic quiescence, an eruption took place in the SW slope of the Cumbre Vieja rift (La Palma, Canary Islands, Spain; Fig. 1), generating a new conical edifice: the Tajogaite volcano (Fig. 2).

The eruption was preluded by a seismic swarm related to magma uprise that was detected at 8–13 km depth by Spain’s National Geographic Institution (IGN by its initials in Spanish), the Canary Islands Volcanological Institution (INVOLCAN) and the Global Volcanism Program^27^. The eruption began at 14:02 GMT in the Tajogaite—Cabeza de Vaca area, which is located ~ 2 km from the El Paso municipality.

Although the composition of the ejected materials changed from tephrite to basanite lava and tephra during the process, the eruption has been classified as a basaltic fissure type, dominated by strombolian activity and with episodic phreatomagmatic pulses. The event corresponds to VEI-3 on the Volcanic Explosivity Index^23,27^.

The strombolian activity started with the ejection of lava flows and pyroclastic materials in a NW–SE 700-m-long fissure. This initial activity generated an eruptive column composed of H_2_O, SO_2_ and other magmatic volatiles, together with volcanic ash, which reached up to 3 km in altitude^30^.

The Tajogaite eruption built a cone that rose 1131 m above sea level showing six major craters on its top, defining a ~ 560-m-long NW–SE eruptive alignment (Figs. 2, S10). The process generated extensive mantles of falling pyroclastic fragments (Figs. 2, S10) that covered a vast area around the southern sector of La Palma, although the ash plume reached the entire Canary Islands archipelago and beyond.

The calculated ejected volcanic volume is approximately 159,106 m^3^ and the affected surface area is estimated at 1219 ha. The eruption destroyed more than 300 agricultural hectares, 73.8 km of public roads, 1646 buildings and produced the evacuation of more than 7000 people.

## Methods

### Sampling and fieldwork

A set of 73 samples was obtained from 32 different fumarole sectors around the Tajogaite volcanic cone, numbered from 1 to 32 (Figs. 2, S10). Fieldwork was carried out during two different campaigns in February and June 2022. For security and preservation reasons, access to the Tajogaite area was heavily restricted and special permits were required in order to perform the corresponding samplings.

Fumarolic vents were extremely active during fieldwork, emitting significant concentrations of toxic gases and reaching temperatures of more than 700 °C in some points. Thus, fieldwork was carried out using appropriate personal protective equipment (gas mask, gloves, glasses, etc.). In addition, we used a PCE-Iberica portable gas detector model MX6 iBRID to monitor air quality and the environmental concentrations of O_2_, CO_,_ HCl, SO_2_ and H_2_S. For safety reasons, and to complement the data gathered during the samplings, ground and fumarole temperatures were measured with a Crimson T-637 thermometric probe.

### X-ray diffraction (XRD)

Powder X-ray diffraction (XRD) measurements were carried out at the XRD lab of GEO3BCN-CSIC (Barcelona, Spain) using a Bruker D8-A25 diffractometer equipped with a Cu X-ray source (Cu K_α_ radiation, λ = 1.5405 Å) and a LynxEye position sensitive detector. For this purpose, nearly randomly oriented powders were prepared by pulverization of the as-collected mineralization. In the case of samples consisting of thin incrustations, the minerals were removed from the rock matrix by careful scratching. The XRD scans were recorded between 4° and 60° in 2θ with a 0.035° step size and equivalent acquisition times of 384 s. Phase identification was carried out using Bruker’s DIFFRAC.EVA software in combination with the PDF-2 (Powder Diffraction File-2) of the International Centre for Diffraction Data, together with the Crystallography Open Database (COD). Semi-quantitative (SQ) phase analyses were performed using the reference intensity ratio (RIR) method. For this purpose, RIR values available in the PDF-2 database were employed. These SQ analyses were mainly aimed at producing a distribution map of the volatile anions in the fumarole minerals (CO_3_^2−^, F^−^, S/SO_4_^2−^, Cl^−^), as these elements can be directly linked to the temperature stage of the mineralization.

### Field emission scanning electron microscopy (FE-SEM)

Selected samples of fumarolic mineralization were prepared in thin section for their textural and mineralogical study at the Laboratory of Geological and Paleontological Preparation (LPGiP) of the Natural Sciences Museum of Barcelona (Barcelona, Spain). A representative selection of the samples was examined in a field emission scanning electron microscope (FE-SEM) model JEOL JSM-7100 at the Scientific and Technological Centres of the University of Barcelona (CCiT-UB). This FE-SEM system is also equipped with an Oxford Instruments EDS (energy dispersive spectroscopy) detector model Pentafex-INCA, which was used to acquire semi-quantitative analyses of fumarole mineral phases as well as to obtain semi-quantitative compositional maps. General operating conditions were 15–20 kV accelerating voltage and 5 nA of beam current.

### Photo consent

The authors declare that all images presented in this manuscript that could lead to identification of study participants have been used with the consent of all subjects and the corresponding authorization for publishing in Scientific Reports.

## Results

### Description of fumarolic efflorescent patches

**Figure 3:**
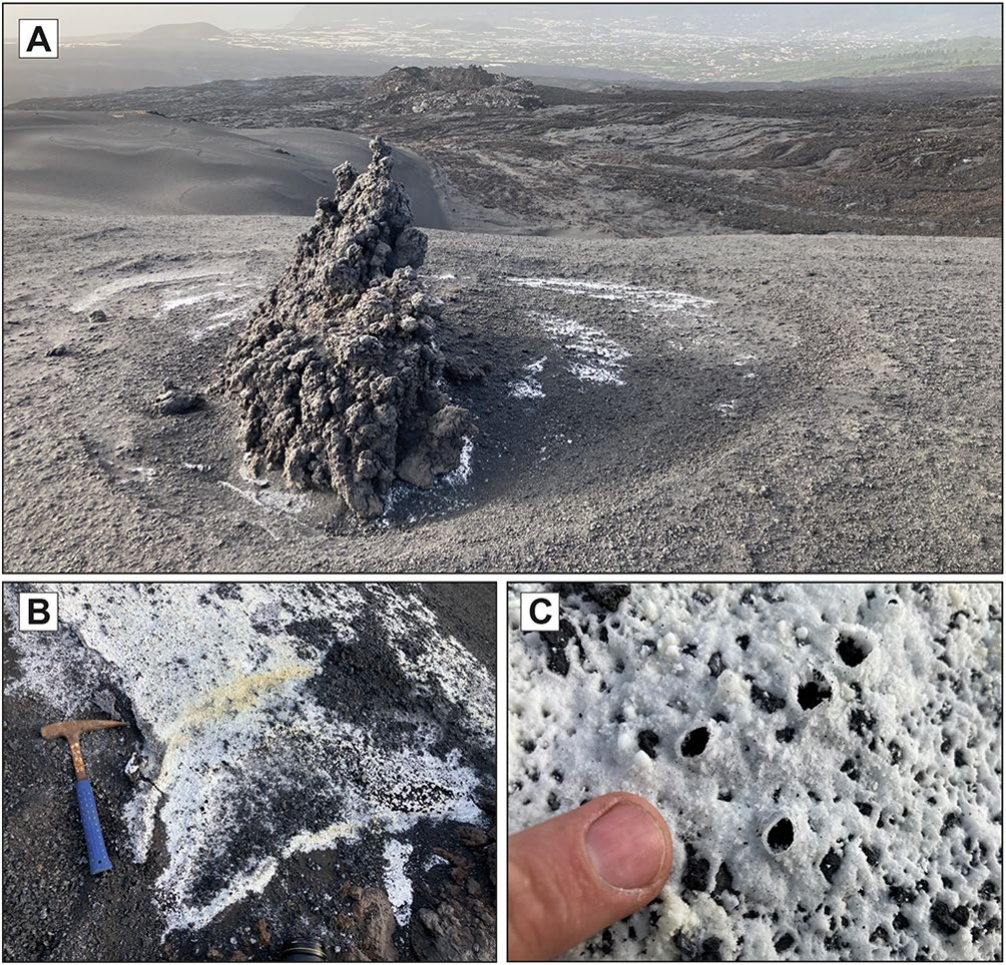
Efflorescent patches associated with the Tajogaite fumaroles located in distal areas of the main craters. (**A**) Whitish patches associated with erratic block porosity, defining a typical concentric distribution. Sampling point 6 (Fig. 2). (**B**) Fumarolic whitish mineralization, mainly composed of carbonates and hydrated sulphates. Sampling point 2 (Fig. 2). (**C**) Detail of image B showing cylindrical microstructures related to gas ejection.

**Figure 4:**
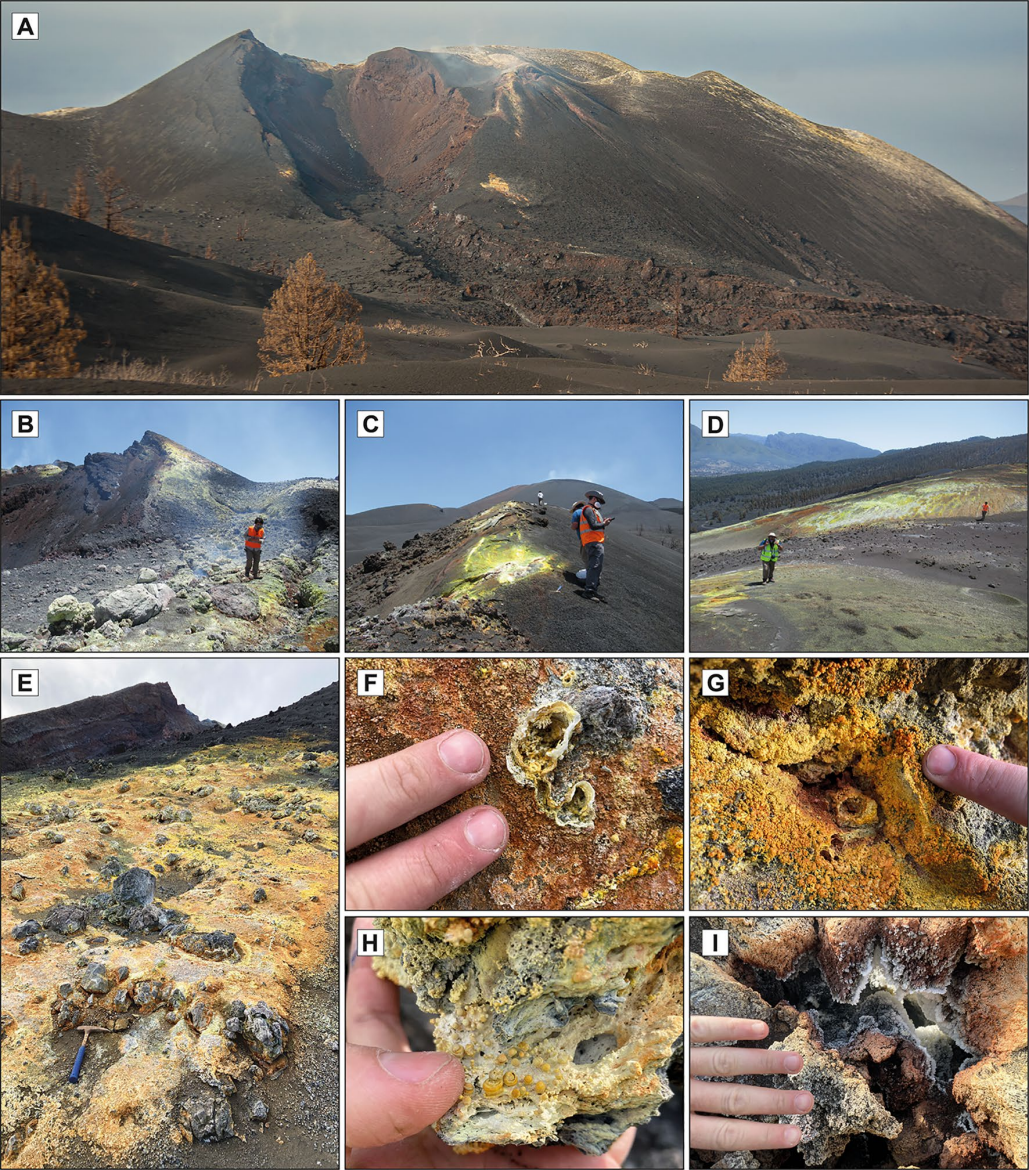
Fumarolic efflorescent patches located in the vicinities of the main craters of the Tajogaite volcano. (**A**) General view of crater “J” (Fig. 2) in which it is possible to distinguish fumarolic efflorescent patches. (**B**, **C**) Fumarolic mineralization formed along secondary fractures with predominance of native sulphur. Sampling points 22 and 23 (Fig. 2). (**D**) Native sulphur mineralization formed on pyroclastic materials due to degassing through porosity and microfractures. Sampling point 9 (Fig. 2). (**D**) General view of typical fumarolic mineralization formed on pyroclastic materials mainly composed of Al–Mg–Ca–Na fluorides and associated chlorides. Sampling point 15 (Fig. 2). (**E–G**) Detail of the concentric morphologies associated with effusive degassing formed by Al–Mg–Ca–Na fluorides. (**H**) Botryoidal aggregates of verneite (Na_2_Ca_3_Al_2_F_14_) formed by the activity of proximal fumaroles located close to the main craters. Sampling point 13 (Fig. 2). (**I**) Whitish aggregates of skeletal microcrystals of Al–Mg–Ca–Na fluorides associated with fumarolic activity on cavities in the volcanic materials. Sampling point 13 (Fig. 2).

In the Canary Islands, fumarolic fields were reported during the historical eruptions of Timanfaya (Lanzarote, 1730–1736)^31,32^, San Juan and Teneguía (La Palma, 1949 and 1971, respectively) as well as in the active crater of the Teide stratovolcano (Tenerife)^33^. In the case of the Tajogaite cone, fumarolic fields associated with degassing were visible in different sectors of the edifice after the end of the main eruptive processes (Fig. 2). As expected, the resulting mineralization generated distinctive efflorescent patches of different colouration, ranging from white to yellow to reddish orange, in clear contrast with the darker volcanic materials (Figs. 3 and 4).

Fumarole deposits located in the distal areas of the main cluster of craters were found to be predominantly whitish. This type of deposit was found to mainly occur by degassing associated with porosity and instability fractures related to erratic blocks which were partially covered by pyroclastic materials (Fig. 3a). This mineralization formed concentric efflorescent patches, where it was possible to distinguish cylindrical microstructures associated with the effusive emission of gases (Fig. 3b,c).

In more proximal fumarolic areas (close to the main cluster of craters), the fumarolic mineralization showed characteristic yellowish-to-reddish orange colourations (Fig. 4a). These efflorescent patches were generally located along secondary degassing fractures (Fig. 4b,c), as well as in small degassing sectors generated through porosity and microfractures of pyroclastic materials (Fig. 4d,e). Precipitated minerals defined circular to conical shapes associated with the effusive degassing (Fig. 4f,g). They were found to be composed of concentric botryoidal formations of yellowish-orange tones (Fig. 4h) as well as skeletal microcrystalline aggregates of variable colouration developed in cavities (Fig. 4i).

### Mineralogy of fumaroles

The mineralogical characterisation of the Tajogaite fumarole samples revealed a very complex mineral assemblage formed by different episodes of overprinting. Due to the strong predominance of cryptocrystalline phases, we considered powder XRD to be a particularly well-suited technique to identify this kind of mineralization. In the supplementary material, selected XRD scans for the studied samples are presented (Figs. S1–S9). In addition, in order to shed some light on the complex paragenesis of the fumaroles, it was necessary to perform textural studies through FE–SEM–EDS analyses of the samples.

**Figure 5:**
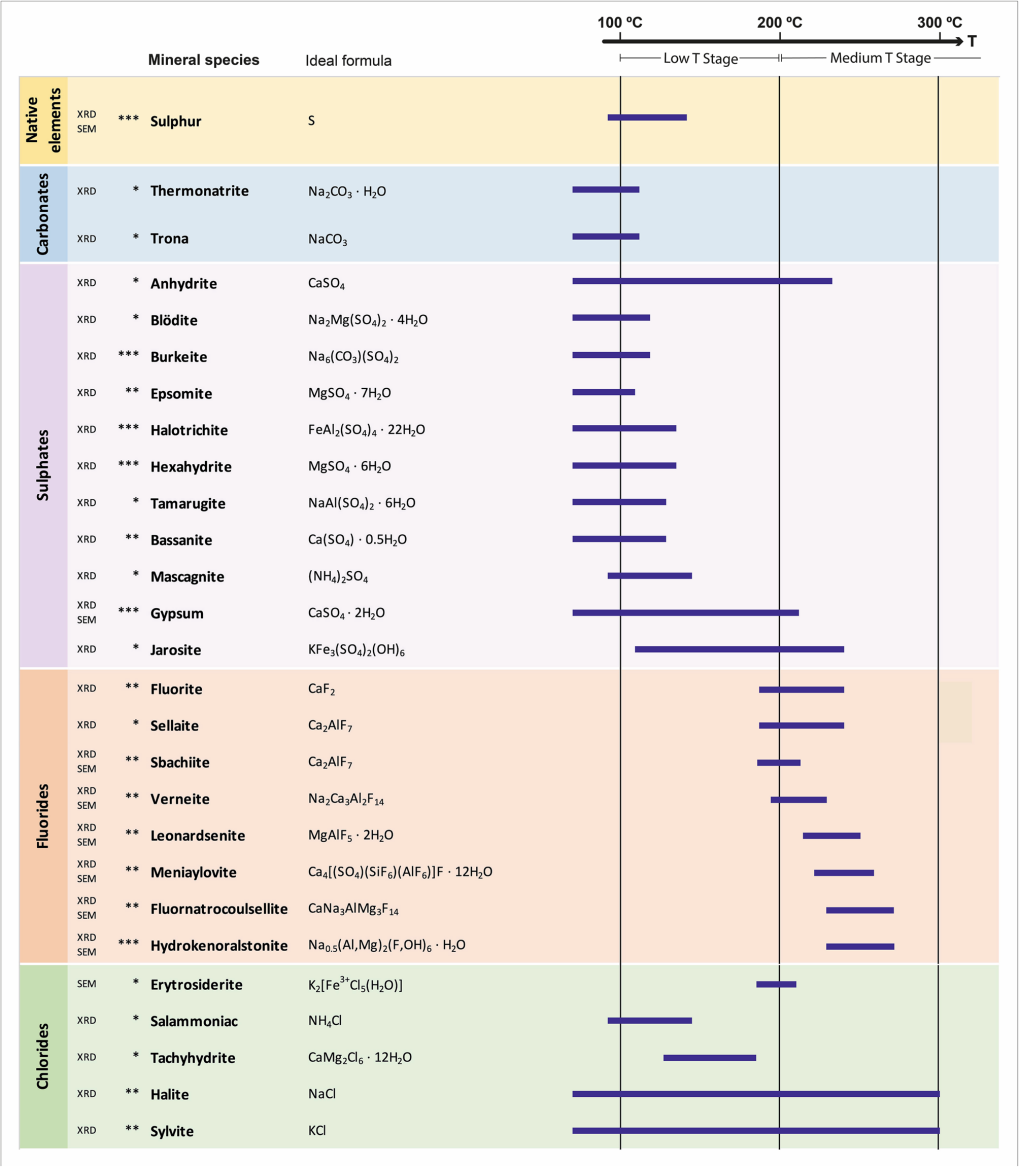
Mineral paragenesis of the Tajogaite fumaroles in relation with temperature. Occurrence of mineral phases: *rare; **common; ***very common. It is also indicated whether species identification was carried out through powder X-Ray diffraction (XRD) or FE–SEM–EDS (SEM).

Recently, Balić-Žunić et al.^34^ proposed a classification of fumarolic phases according to mineralization temperatures, distinguishing mineral paragenesis of (1) high temperature (HT, > 400 °C), (2) medium temperature (MT, 200–400 °C), and (3) low temperature (LT, < 200 °C). A plausible mineral paragenesis for the Tajogaite fumaroles in relation to these temperature ranges is proposed in Fig. 5 and discussed in more detail in the following subsections.

#### Native elements

**Figure 6:**
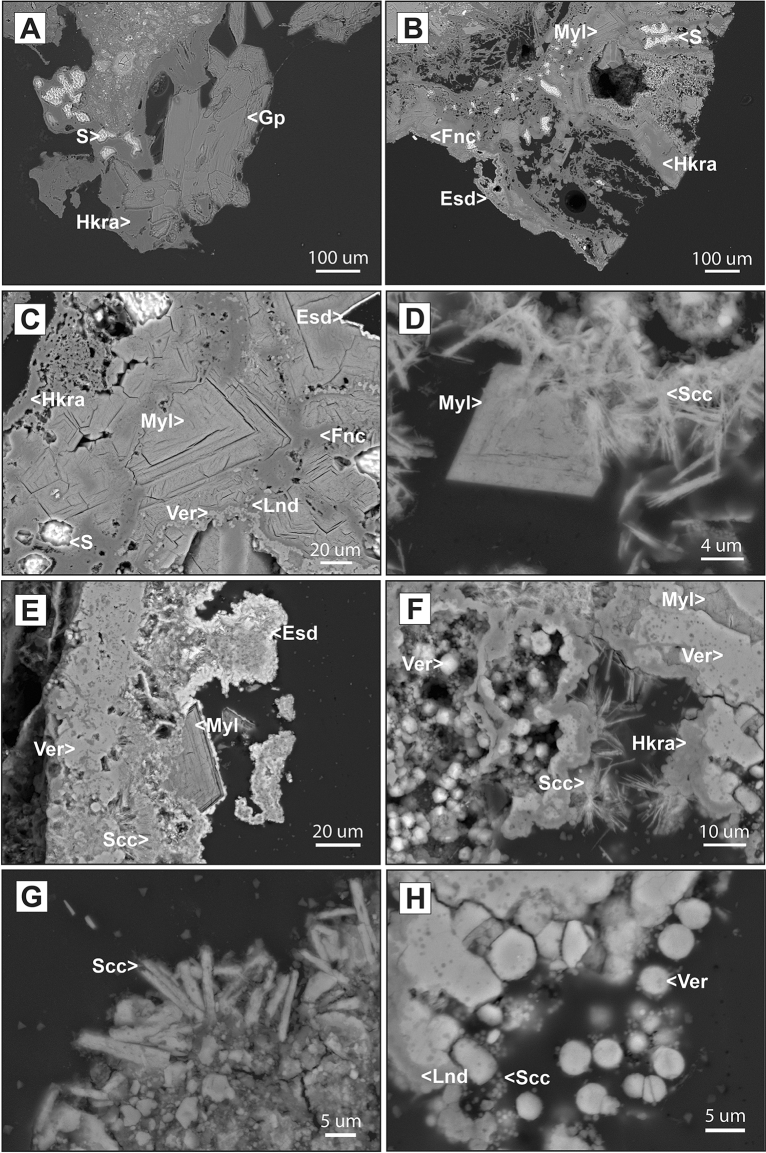
SEM (backscattered electron, BSE) images of the Tajogaite fumarolic mineralization. (**A**) Euhedral crystals of gypsum (Gp) associated with hydrokenoralstonite (Hkra) and skeletal crystals of native sulphur (S). (**B**) Skeletal crystals of native sulphur (S) typically associated with Al–Mg–Ca–Na fluorides: hydrokenoralstonite (Hkra), fluornatrocoulsellite (Fnc) and meniaylovite (Myl) covered by a later patina of erytrosiderite (Esd). (**C**) Complete Al–Mg–Ca–Na fluoride assemblage of the Tajogaite area: hydrokenoralstonite (Hkra), leonardsenite (Lnd), verneite (Ver) and fluornatrocoulsellite (Fnc) with the typical later patina of erytrosiderite (Esd). (**D**) Euhedral crystal of meniaylovite (Myl) associated with acicular aggregates of sbacchiite (Scc). (**E**) Erytrosiderite (Esd) covering and euhedral crystal of meniaylovite (Myl) associated with verneite (Ver) and acicular crystals of sbacchiite (Scc). (**F**) Globular aggregates of verneite (Ver) associated with leonardsenite (Lnd) and later sbacchiite (Scc). (**G**) Detailed view of acicular aggregates of sbacchiite (Scc). (**H**) Typical fluorides assemblage composed of hydrokenoralstonite (Hkra), botryoidal verneite (Ver), meniaylovite (Myl) and later acicular crystals of sbacchiite (Scc).

Among the group of native elements, sulphur is the only species reported in the fumaroles of the Tajogaite volcano (Figs. 5, S1). Native sulphur is one of the most common minerals in fumaroles worldwide (Table S1). In Tajogaite, it occurs as well-defined skeletal crystals up to 2 cm in size, developed in small cavities and irregular surfaces and generating highly characteristic lemon-yellow patches (Fig. 4a–d). Although sulphur formation is related only to low temperature conditions (< 120 °C)^34^, it can be found associated with a significant range of species due to the evolution of thermal conditions and overprinting with different mineral stages. In more proximal fumarolic areas to the craters’ alignment, it is commonly associated with Al–Mg–Fe–Ca fluorides such as hydrokenoralstonite, fluornatrocoulsellite or meniaylovite (Fig. 6a–c).

#### Carbonates

Due to the typical acid conditions of fumarolic environments, carbonates are unusual phases not only in the Tajogaite fumaroles but also in most fumarolic localities worldwide (Table S1). In Tajogaite, we only identified two species of alkaline carbonates (thermonatrite and trona; Figs. 5, S2–S3) with XRD. These minerals form typical cryptocrystalline millimetric crusts. Both carbonates were only identified in distal fumaroles in association with hydrated sulphates, forming typical whitish efflorescent patches (Fig. 3).

#### Sulphates

Sulphates are the most represented group in the Tajogaite fumaroles. In fact, up to 10 species were identified in the volcano fumaroles (Fig. 5). Sulphates were generally found in more distal fumaroles, generating typical whitish patches commonly associated with halite and alkaline carbonates (Figs. S1–S4). Only a few species of sulphates (e.g., gypsum, mascagnite, anhydrite and jarosite) occurred in orange-yellowish patches located more proximal fumaroles, where they are typically associated with Al–Mg–Fe–Ca fluorides (Fig. 6a).

#### Fluorides

The group of fluorides is probably the most characteristic and abundant in the orange-yellowish fumarolic patches of the more proximal fumaroles (Fig. 4e–h). In Tajogaite, we identified up to 8 different species of Al–Mg–Fe–Ca fluorides (fluorite, sellaite, fluornatrocoulsellite, hydrokenoralstonite, meniaylovite, leonardsenite, verneite and sbachiite, Figs. 5, S5–S8). Other unidentified compounds are likely present in these samples. A very similar assemblage has been reported in fumarolic localities from Iceland (e.g., Eldfell, Hekla and Surtsey; Table S1). Although these fluorides normally occur as massive cryptocrystalline aggregates (e.g., fluornatrocoulsellite, hydrokenoralstonite or leonardsenite), it is possible to distinguish well-defined octahedral crystals of meniaylovite (Fig. 6c–e), generally associated with acicular sbachiite (Fig. 6f,g). Verneite also occurs as botryoidal aggregates (Fig. 6h) that can reach up to 3 mm and are observable with the naked eye (Fig. 4h).

#### Chlorides

Chlorides are well represented in different areas of the Tajogaite fumaroles. One of them is salammoniac, which is a very common species in several localities worldwide (Table S1) but relatively scarce in the Tajogaite area. It was only identified by XRD associated with native sulphur (Fig. S9) in proximal fumarolic areas. The rest of the identified chlorides (halite, sylvite, thermonatrite and erytrosiderite) are typically found in association with the Al–Mg–Fe–Ca fluorides assemblage (Fig. S5). In fact, erytrosiderite is generally related to the last stage of crystallization of the Al–Mg–Fe–Ca fluorides since it normally occurs as a fine micrometric patina that covers the fluoride assemblage (Fig. 6e).

## Discussion

### Geomorphological control of the location of fumaroles

The location of fumaroles and gas-steam vents (common in geothermal fields) marks tectonically weakened zones and is directly controlled by terrain permeability. In turn, the permeability of volcanic edifices is related to the regional and local tectonic framework, which determines the direction of the fractures through which gas can easily reach the surface^35,36^.

In the CISP, the tectonic setting corresponds to an intraplate passive margin. Thus, notable fracture zones due to plate motion are absent and regional historical seismicity is only controlled by volcanic activity^37^. Nevertheless, fractures generated by the subsidence of eruptive materials and the corresponding terrain instability are frequent in the Canary Islands^38–41^.

Island rifts are another typical geomorphological feature associated with terrain fractures in oceanic volcanic islands and are widespread in the CISP. These ridges are formed by parallel and subparallel dike swarms generated by magma migration from successive eruptions with nearby magmatic sources through normal faults^42,43^. A significant strain zone is formed beneath the rift axis due to the horizontal gravitational stress induced by the topography^44^. This instability path is well used by magma and associated gases in their ascent. Models made of gelatine blocks have successfully reproduced this behaviour in laboratory experiments^45,46^.

In the case of the Tajogaite volcano, we observe that the craters are aligned in an NNW direction close to the axis direction of the Cumbre Vieja rift (Figs. 1, 2 and S10), and seismic records show magma migration from south to north following this rift direction^47^. A similar topography-induced gravitational stress effect has been described at the scale of crater rims^36,48^. Concentric faults and associated fumaroles follow the Tajogaite crater rim, evidencing that the stress field also plays a topographic control on fracture lineation at local scale (Figs. 2, S10).

In addition, we report two ridges with significant fumaroles on the top, located to the SW of the volcanic cone cluster (Fig. 2). They correspond to the relict distal part of an elongated crater rim opened to the west during the first eruptive stage (circa 27th September 2021).

At this local scale we speculate that, in addition to the effect of topography-induced gravity stress, the effect of gravity control on the thickness of the volcanoclastic cover should be added. The slope of crater flanks and ridges may also control the thickness of the tephra cover deposited on the surface. Accordingly, the thickness of the volcanoclastic sediments would be thinner on the top of the ridges and the crater rims and thicker in the negative reliefs and the crater centre. Therefore, this control in the thickness of the volcano-sedimentary deposits would produce more permeability to gas circulation in the elevated reliefs, explaining the major occurrence of fumaroles in these areas of the Tajogaite volcano (Fig. 2).

### Distribution of anions in the fumarolic mineralization

With the aim of evaluating the degree of compositional homogeneity and the mineralization temperatures in the fumarolic field of the Tajogaite volcano, we performed a rough estimation of the content of the following major cations/anions in the fumarole minerals: CO_3_^2−^, F, Al, Na, Mg, Al, S, SO_4_^2−^, Cl, Ca and Fe. For this purpose, we employed SQ data obtained from SQ phase analyses of the XRD scans using the RIR method or, in some cases, from the SEM–EDS analyses. In the former case, using the weight per cent values obtained for the identified minerals in each sample and neglecting the contribution of volcanic phases and alteration minerals like Fe oxides, we qualitatively inferred the major elements in the investigated samples. These data allowed us to produce a mineralogical map showing a tentative distribution of the most abundant anionic elements in the fumarole minerals [namely C (in the form of carbonates), F (in the form of fluorides), S (native sulphur or in the form of sulphates) and Cl (in the form of chlorides)] in the fumarole field of Tajogaite. Although these elements were directly ejected during degassing, it should be noted that here we are not trying to produce a gas composition map. These elements basically correspond to the major compositional anions present in the fumarolic minerals^49^. In particular, we make the distinction between native sulphur and SO_4_^2−^ because we are specifically interested in the connection between the distribution and homogeneity of the mineralization, and also in their formation temperature.

The results thus obtained are plotted in the map of Fig. 2, where it can be seen that anion distribution is, at first sight, fairly inhomogeneous. However, closer inspection suggests that the low temperature native sulphur assemblage prevails around the craters’ alignment as well as in proximal zones covered by thick layers of tephra. In contrast, no native sulphur predominance is found in the NE lava flows, where higher-temperature halogen-bearing minerals seem to prevail. In turn, among the investigated samples, low temperature carbonates and sulphates are only found as major phases in the western distal regions. It should be noted that in Fig. 2 we included samples collected in both February and June 2022 as they did not show significant compositional differences, at least from the point of view of the major elements obtained by XRD-RIR or SEM–EDS.

It is well known that HCl and HF are more soluble than SO_2_ in the magma, which might partly explain the observed distribution of minerals in the fumarole fields^49^. According to this interpretation, the most active emanations should have higher content in sulphur gases, as these would tend to escape earlier. Gas emanations would then be enriched in Cl and F at later degassing stages, and therefore also in more distal zones like the lava flows. However, this interpretation does not take into account the complex dynamics of degassing, which depends on numerous factors like gas concentration, temperature and pressure, the possible extent of ground-water dilution, or the interactions between the emanations and the wall rock. Indeed, elevated CO_2_/S ratios might be diagnostic of intermediate pressures (100–300 MPa), while lower pressures could imply high H_2_O/CO_2_ and S/Cl ratios^50^. According to the two-stage degassing model of Pennisi and Le Cloarec^51^, enriched Cl/S ratios would correspond to deep degassing, while sulphur enrichment would be associated with ambient pressure (shallow) degassing. Regardless of pressure, it is clear that, as discussed above, halide mineralization necessarily takes place at higher temperatures, as in the case of sampling point 15 (Fig. 2). In contrast, areas around the rim of the volcano, at much lower temperatures, give rise to large extensions of sulphur-bearing incrustations. In addition, it cannot be ruled out that sea water plays some role in the fumarolic mineralization of this type of eruption, which would strongly affect the final distribution of halide phases^52^.

### Fumarolic mineral sequence

Since the beginning of the twentieth century, attempts have been made to establish a relation between the genetic conditions of volcanic fumaroles and the mineral assemblage formed in these environments^53–55^. As previously mentioned, one of the most recent proposals is the temperature-related mineral classification of Balić-Žunić et al.^34^, which distinguishes mineral assemblages of (1) high temperature (HT, > 400 °C), (2) medium temperature (MT, 200–400 °C), and (3) low temperature (LT, < 200 °C). However, the same authors suggest that minerals of these three groups may occur closely associated, which can be attributed to the significant fluctuation of temperature conditions that takes place in volcanic environments.

In the case of the Tajogaite fumaroles, mineralogy strongly reflects this overprinting pattern. However, at least three different mineral assemblages may be clearly identified, related to specific thermal conditions of formation.

**Figure 7:**
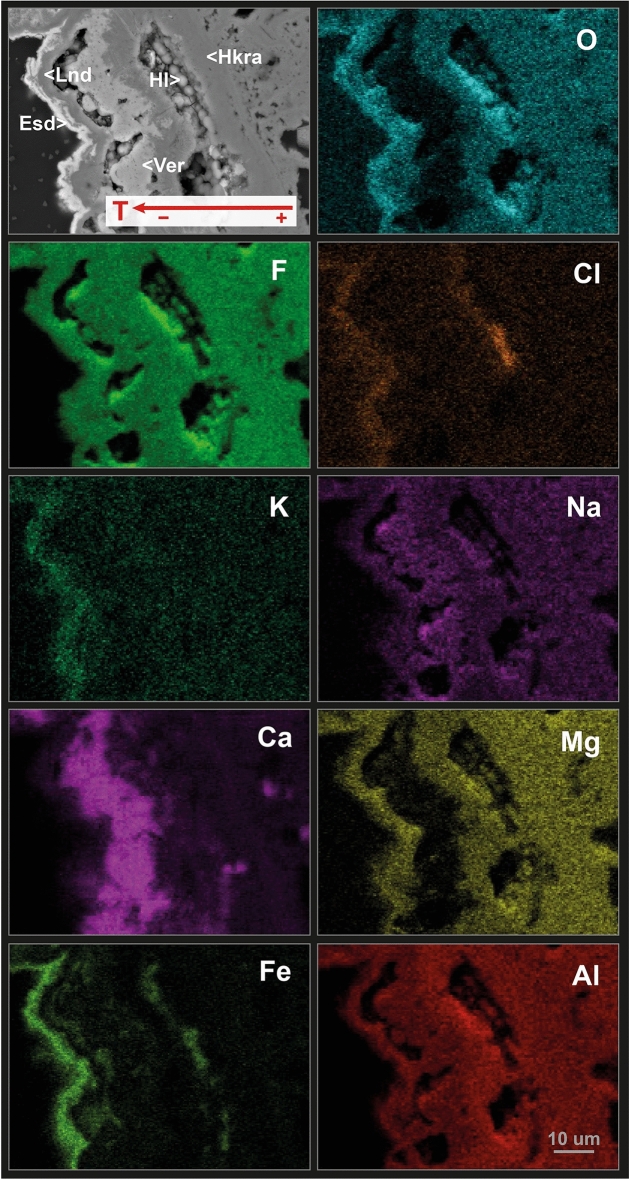
Wavelength-dispersive X-ray maps of representative elements of the typical fluoride-chloride assemblage from the Tajogaite proximal fumaroles: erytrosiderite (Esd) leonardsenite (Lnd), verneite (Ver), halite (Hl) and hydrokenoralstonite (Hkra).

The first typical assemblage is especially found in more proximal fumarolic areas. They form yellowish-orange patches (Fig. 4e–i), mainly composed of Al–Mg–Fe–Ca fluorides (e.g., hydrokenoralstonite, fluornatrocoulsellite, meniaylovite, verneite, leonardsenite, and sbachiite) commonly associated with chlorides. The mineralogical and textural study of this second type of sample revealed a well-established crystallization sequence. During higher temperature conditions hydrokenoralstonite is the most abundant mineral phase (Fig. 7) and is generally accompanied by fluornatrocoulsellite and meniaylovite (Fig. 6b,c). A second cooler stage produced a Ca enrichment and the formation of verneite (Fig. 7), which is followed by the crystallization of leonardsenite (Fig. 7). Sbachiite is present in some samples as well-defined acicular crystals corresponding to the later stage of formation (Fig. 6f). The sequence is typically characterized by enrichment in Fe (Fig. 7) and the formation of a micrometric erytrosiderite patina, which was identified in almost all the studied samples (Figs. 6b,e, 7). According to the classification proposed by Balić-Žunić et al.^31^, this assemblage should be assigned to MT conditions (200–400 °C). However, alkaline chlorides (e.g., halite and sylvite) generally represent the highest temperature products and the thermal limit of formation should be then established around 300 °C (Fig. 5).

In certain sectors of these MT fumarolic areas, the mineralization of fluorides and chlorides is commonly accompanied by a second assemblage that is characterized by the prevalence of native sulphur, a mineral phase classically related to LT conditions (< 120 °C)^31^. In addition, native sulphur could also be associated with other LT phases such as gypsum (Figs. 6a, S1), mascagnite and salammoniac (Figs. 5, S9). The observation of occasional LT minerals in association with the MT phases is likely a consequence of significant temperature variations in the fumarole environment, and consequently in the entire volcanic system.

The third assemblage is characteristic from vents located in distal zones, but also in the external part of proximal fumaroles. This type of mineralization defines white efflorescent patches (Fig. 3a–c), mainly composed of hydrated sulphates (e.g., gypsum, halotrichite, hexahydrite, epsomite or tamarugite), frequently associated with alkaline carbonates (e.g., thermonatrite, trona). It should be noted that native sulphur is absent in this specific mineralization. The widespread occurrence of alkaline carbonates in this type of fumarolic mineralization suggests neutral pH conditions, since carbonates are soluble in high acidity environments. These specific pH conditions may also indicate the circulation of high volumes of liquid water, suggesting that this mineral assemblage may be related to LT conditions, necessarily under 100 °C (Fig. 5). This mineral association is easily achievable by overprinting of crystallizing phases during the corresponding cooling of volcanic materials after the main eruptive process. The fact that this mineral sequence is not found beyond the vicinities of the main cluster of craters may be related to temperature fluctuations in the fumarolic system that can generate more heterogeneity and a chaotic overprinting.

In the recent work by Martínez-Martínez et al.^11^, a general preliminary classification of the Tajogaite fumarolic mineralization has been proposed on the basis of gas measurements and field work performed only one month after the end of the active volcanic period. These authors report: (1) a sulphur-sulphate zone, proximal to the area of the craters; (2) a halide zone and (3) a salammoniac zone in medium-distal areas. This conclusion is, in general terms, similar and congruent to the model proposed in the present manuscript, where we present data with higher spatial resolution but only around the volcanic edifice. While Martinez-Martinez and co-authors report many interesting instances of (NH_4_)-bearing minerals, in the present work we have found a larger number of species, like Al–Mg–Fe–Ca fluorides, unambiguously identified by petrographic observations and XRD measurements. The different sampling periods and locations, together with the complexity of this type of field campaigns, may partly explain the observed differences, thus suggesting that similar future works might benefit from samplings with much higher temporal and spatial resolutions.

Martínez-Martínez et al.^11^ also conclude, on the basis of on-site temperature measurements, that these mineral associations may occur at much higher temperatures than those reported in previous works. This conclusion also deserves future research. According to our own measurements and in-situ observations, the determination of fumarolic temperatures, which may be strongly affected by uncontrolled factors like wind conditions or wall rock heat diffusion and cooling, is not straightforward and might be easily overestimated. Experimental set-ups like those proposed by Zelenski et al.^56^ may provide invaluable information about the specific gas composition and temperature required to precipitate fumarolic minerals.

**Figure 8:**
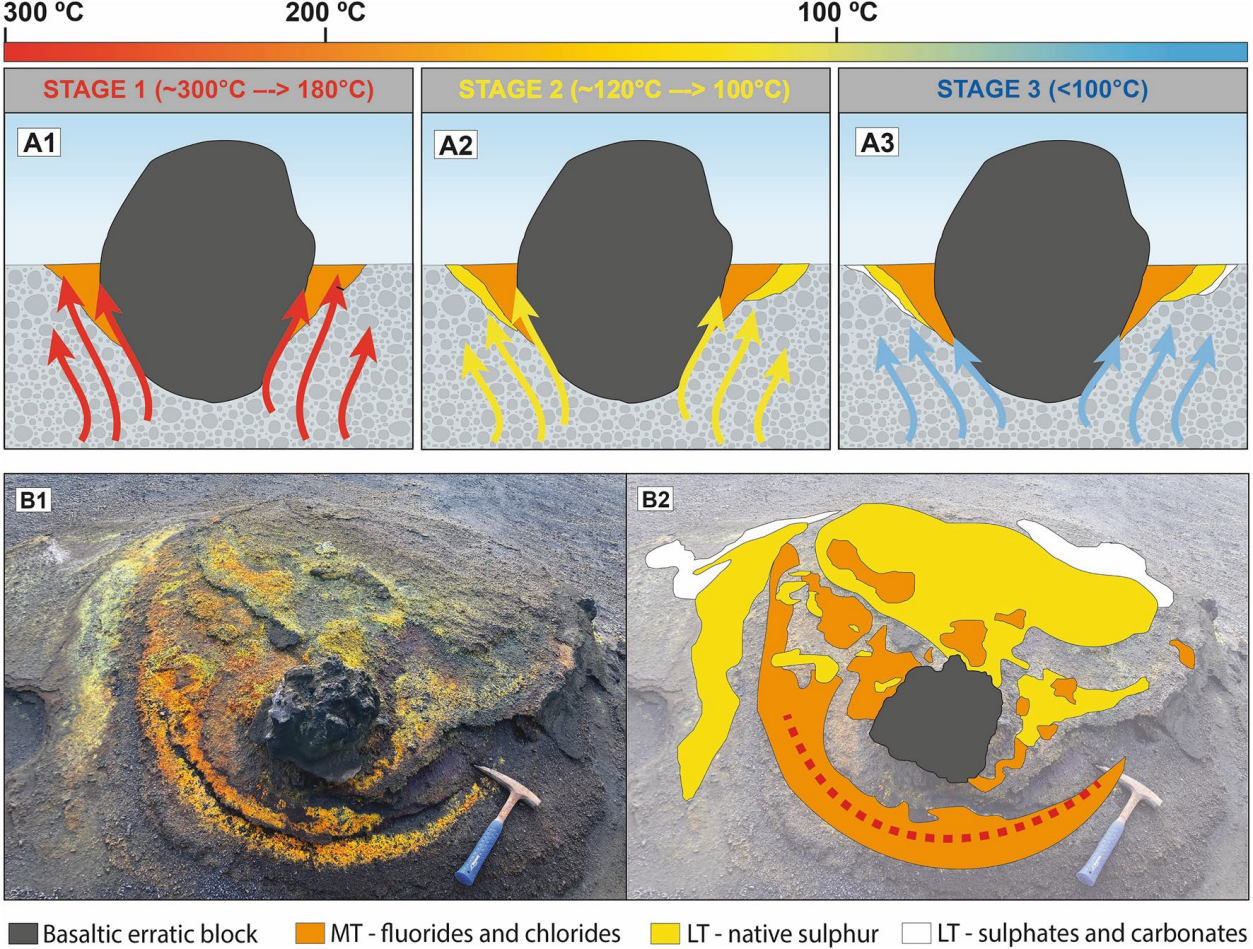
(**A**) Schematic model of the formation of the Tajogaite fumarolic mineralization according to different stages of cooling. (**B**) General view of a fumarolic outcrop developed around an erratic block. It is possible to distinguish the typical mineral sequence developed during cooling in Tajogaite, with occurrence of mineral assemblages of (1) medium temperature (MT) fluorides and chlorides (~ 300–180 °C); (2) low temperature (LT) native sulphur in association with other accessory phases (e.g., gypsum, mascagnite and salammoniac) (~ 120–100 °C), and lately (3) low temperature (LT) sulphates and alkaline carbonates related with the lowest temperature conditions (< 100 °C).

Finally, it is interesting to note that our model is nicely reproduced at a much smaller scale in some fumaroles around erratic blocks, which were not found to involve very high temperatures. As can be seen in Fig. 8, the three characteristic assemblages produced as a function of decreasing temperature are easily identified: (1) the medium temperature Al–Mg–Fe–Ca fluorides and associated chlorides close to the erratic blocks (~ 300–180 °C); (2) native sulphur associated with gypsum, mascagnite and salammoniac (~ 120–100 °C) at somewhat more distant (and therefore cooler) points around the block, and (3) low temperature hydrated sulphates and carbonates related to still cooler areas (< 100 °C).

## Conclusions

Despite their clear interest both from a mineralogical and volcanological point of view, detailed mineralogical studies of fumaroles are globally scarce. The present work provides a good example of how detailed mineralogy can provide significant information about the thermal evolution in this kind of environment. For instance, it seems clear that continuous monitorization of fumarole mineralization could provide a better understanding of the evolution of the volcanic system in question and could be useful to complement volcanological studies. The final aim of such surveying tools is to provide as much information as possible with a view to management of the associated volcanic risk.

In summary, the main conclusions of this work are as follows:

1. Mineralization related to the Tajogaite fumaroles formed efflorescent patches located at variable distance from the main cluster of volcanic craters. Distal patches are predominantly whitish, while in the vicinities of the main craters, they typically showed yellowish to orange colours.
2. The location of the Tajogaite fumaroles is directly controlled by the permeability of the volcanic materials. Fumaroles mainly occur at elevated topographic areas which are more permeable to gas circulation.
3. A complex mineral assemblage has been described in the Tajogaite fumaroles. It is dominated by phases related to low (< 200 °C) and medium temperature (200–400 °C) conditions.
4. Three different types of mineralization have been distinguished: (1) Al–Mg—Fe–Ca fluorides and associated chlorides located at proximal fumaroles (~ 300–180 °C); (2) native sulphur and associated LT phases (e.g., gypsum and, more rarely, mascagnite and salammoniac), related to cooler stages of the proximal mineralization (~ 120–100 °C); (3) sulphates and alkaline carbonates associated with distal fumarolic areas (< 100 °C).
5. The observation of LT minerals in association with MT phases is likely a consequence of the variation of temperature of the fumarole environment. However, more research should be performed to fully understand how the different mineral assemblages depend on temperature as well as on other key parameters (gas composition, pH, pressure, rock permeability, etc.) in order to link the evolution of the volcanic system with these reported fumarolic mineralization.

## Supplementary Information

Supplementary Information.

## Data Availability

The authors confirm that the data supporting the findings of this study are available within the article and its supplementary materials.
