# Supplementary material for: The ephemeral fumarolic mineralization of the 2021 Tajogaite volcanic eruption (La Palma, Canary Islands, Spain)
Source: Sci Rep. 2023 Apr 18;13:6336. doi: 10.1038/s41598-023-33387-6 (PMC10113252; doi:10.1038/s41598-023-33387-6)
Supplement: Supplementary file 1 — Supplementary Information. [file 41598_2023_33387_MOESM1_ESM.pdf]

SUPPLEMENTARY MATERIAL

The ephemeral fumarolic mineralization of the 2021 Tajogaite volcanic eruption (La Palma, Canary Islands, Spain)

Marc Campeny[1*], Inmaculada Menéndez[2]; Jordi Ibáñez-Insa[3], Jesús Rivera-Martínez[4], Jorge Yepes[2], Soledad Álvarez-Pousa[3], Jorge Méndez-Ramos[5], José Mangas[2]

[1] Departament de Mineralogia, Museu de Ciències Naturals de Barcelona, 08003 Barcelona, Spain
[2] Instituto de Oceanografía y Cambio Global, IOCAG, Universidad de Las Palmas de Gran Canaria, 35214, Telde, Las Palmas de Gran Canaria, Spain
[3] Geosciences Barcelona (GEO3BCN), Spanish Council for Scientific Research (CSIC), Lluís Solé i Sabarís s/n, 08028 Barcelona, Spain
[4] Spanish Oceanographic Institute, Servicios Centrales, C/ C. de María 8, 28002 Madrid, Spain
[5] Departamento de Física, Universidad de La Laguna, 38206 La Laguna, Tenerife, Spain

***** Correspondence: mcampenyc@bcn.cat

This file includes:

- Figures S1 to S9: **Powder X-Ray Diffraction Measurements**
- Figure S10: **Tajogaite volcano geological map**
- Table 1: **Mineralogy of different fumarolic localities worldwide**

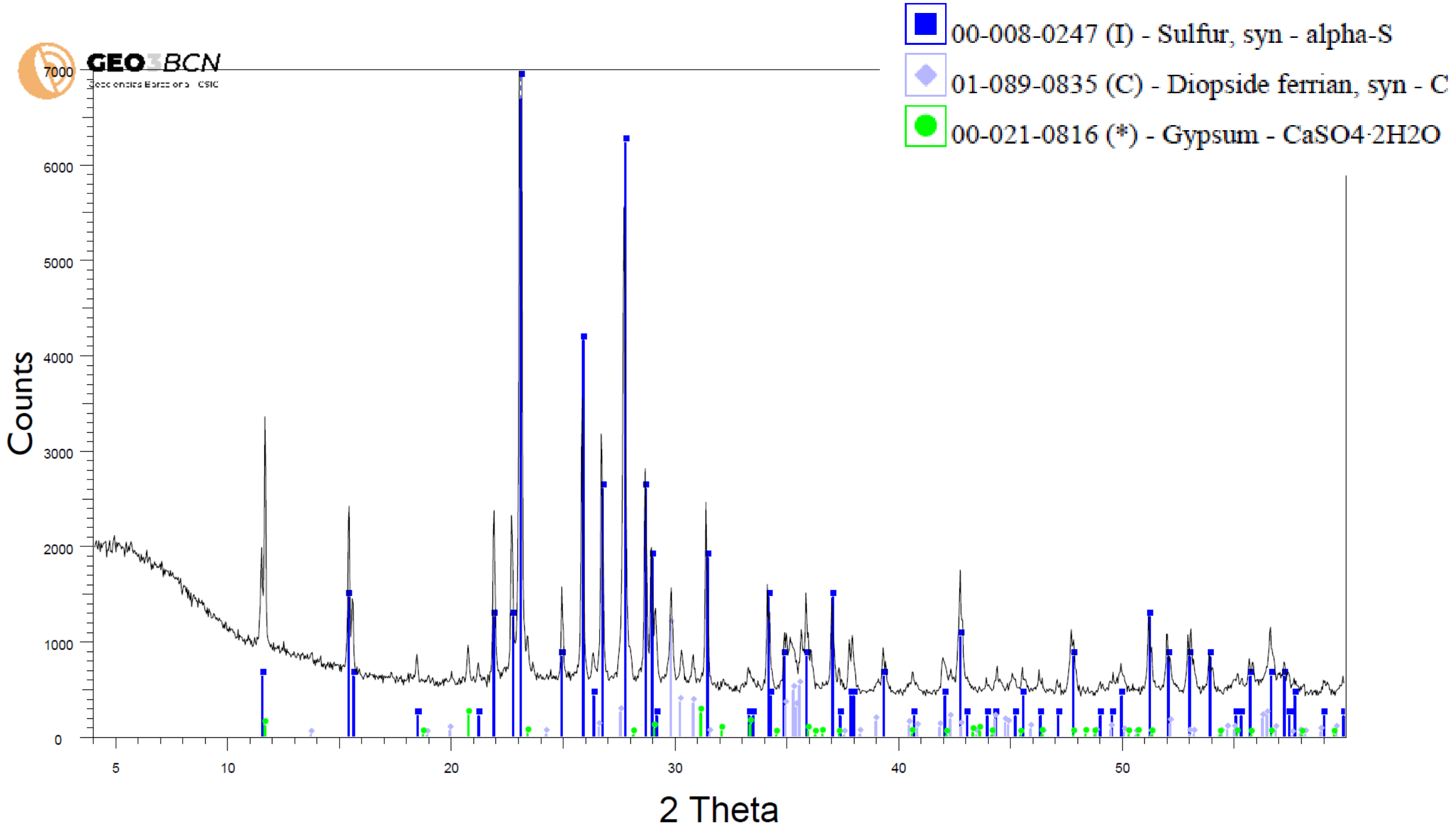

Figure S1: Powder x-ray diffraction scan of sample Ref. LP_E_F_i, collected in the sampling point number 9 (see Figures 2 and 3). The scan is clearly dominated by the peaks of native sulphur (orthorhombic α-sulphur) and weaker peaks of gypsum. Minor magmatic diopside is also show up in the scan.

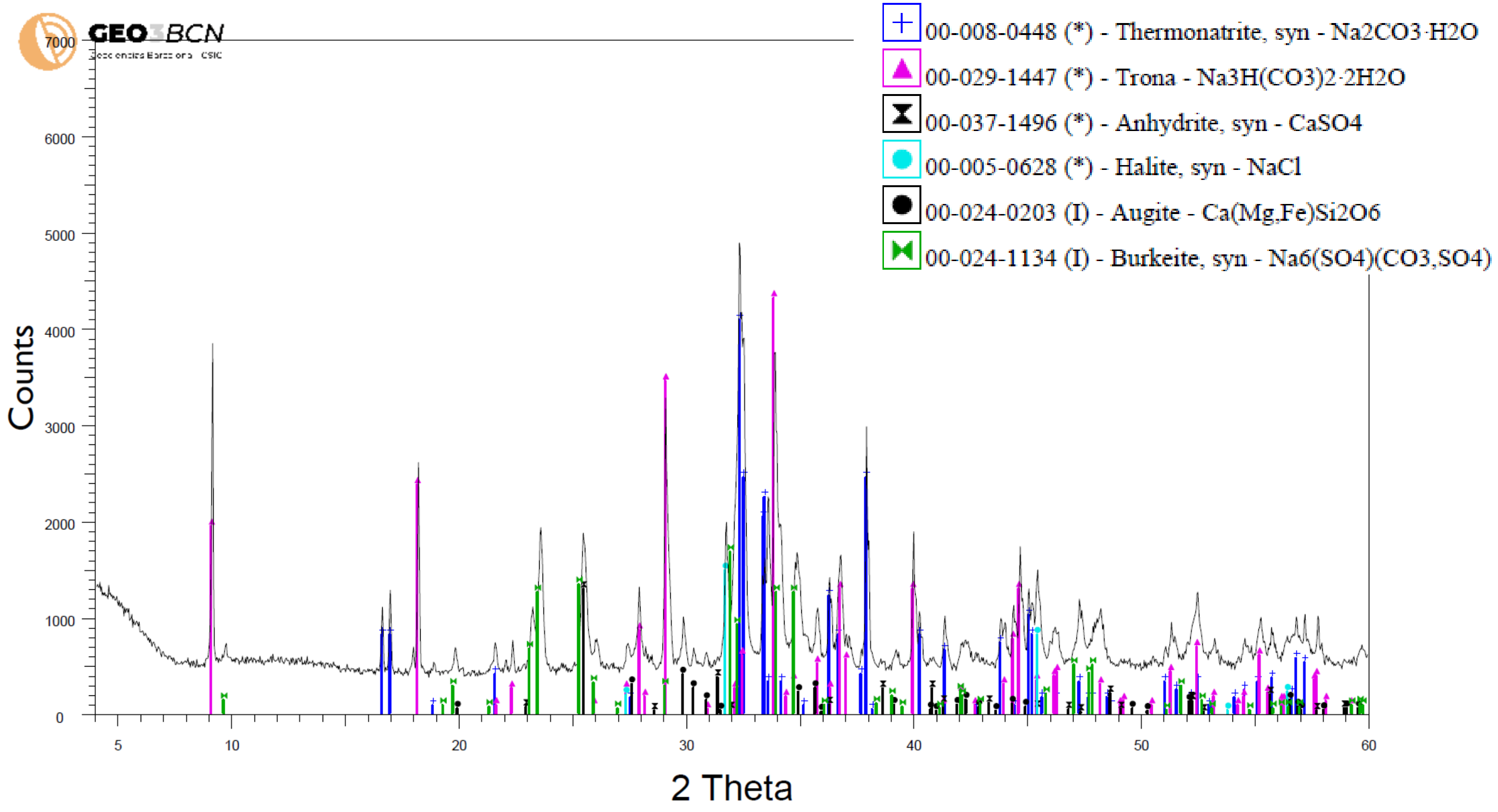

Figure S2: Powder x-ray diffraction scan of sample Ref. LP_E_F_d, collected in the sampling point number 4 (see Figures 2 and 3). The scan allows identifying several fumarolic mineral phases: thermonatrite, trona, anhydrite, burkeite and halite. The peaks of burkeite are sizably shifted in relation to the PDF-2 pattern, which can be attributed to stoichiometric differences between the mineral in the collected samples and the phase used to obtained the pattern of the PDF-2 database.

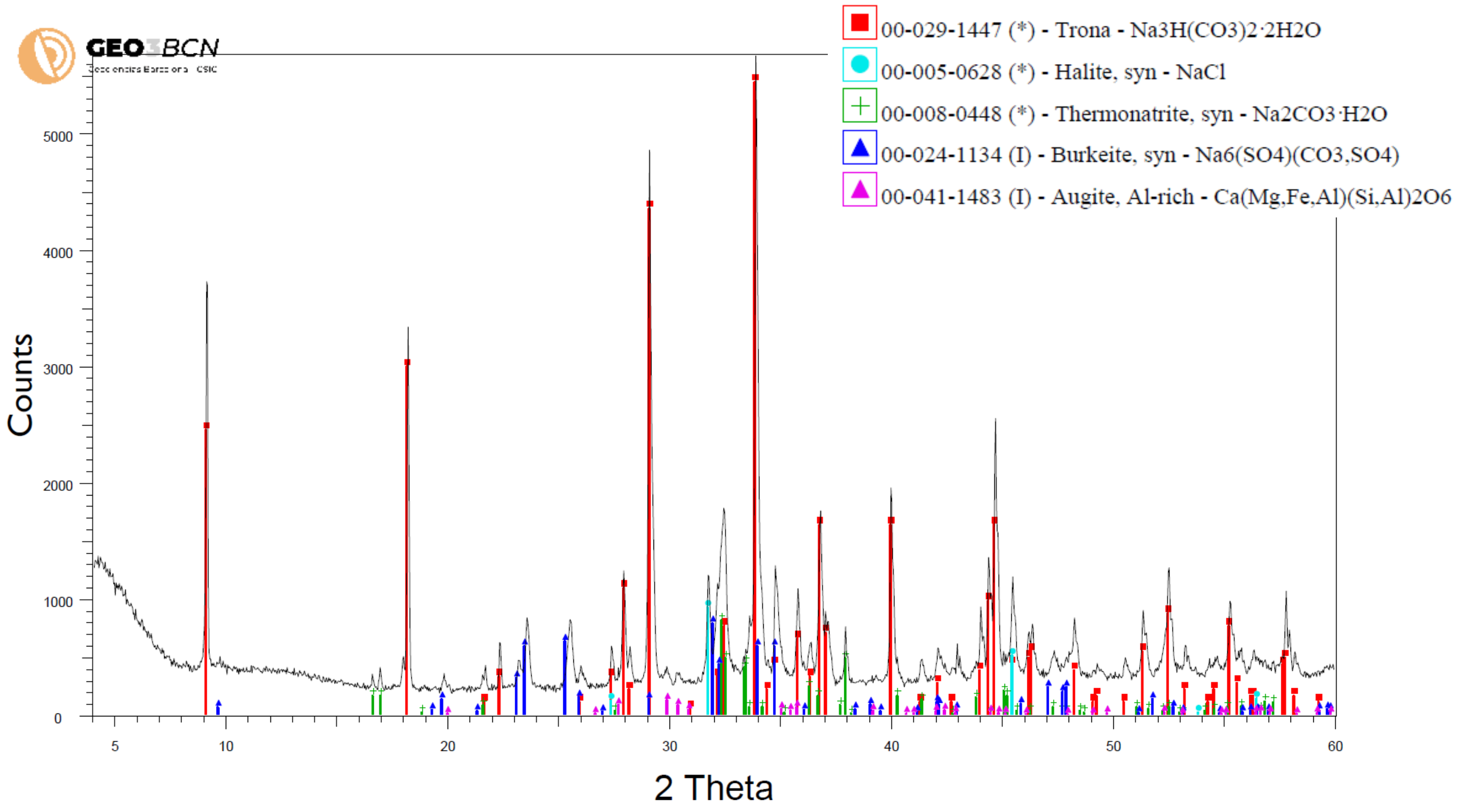

Figure S3: Powder x-ray diffraction scan of sample Ref. LP_E_F_c, collected in the sampling point number 3 (see Figs 2 and 3). The scan is clearly dominated by the peaks of trona. The rest of weaker features that show up in the scan can be assigned to halite, thermonatrite, and burkeite as well as to small amounts of augite.

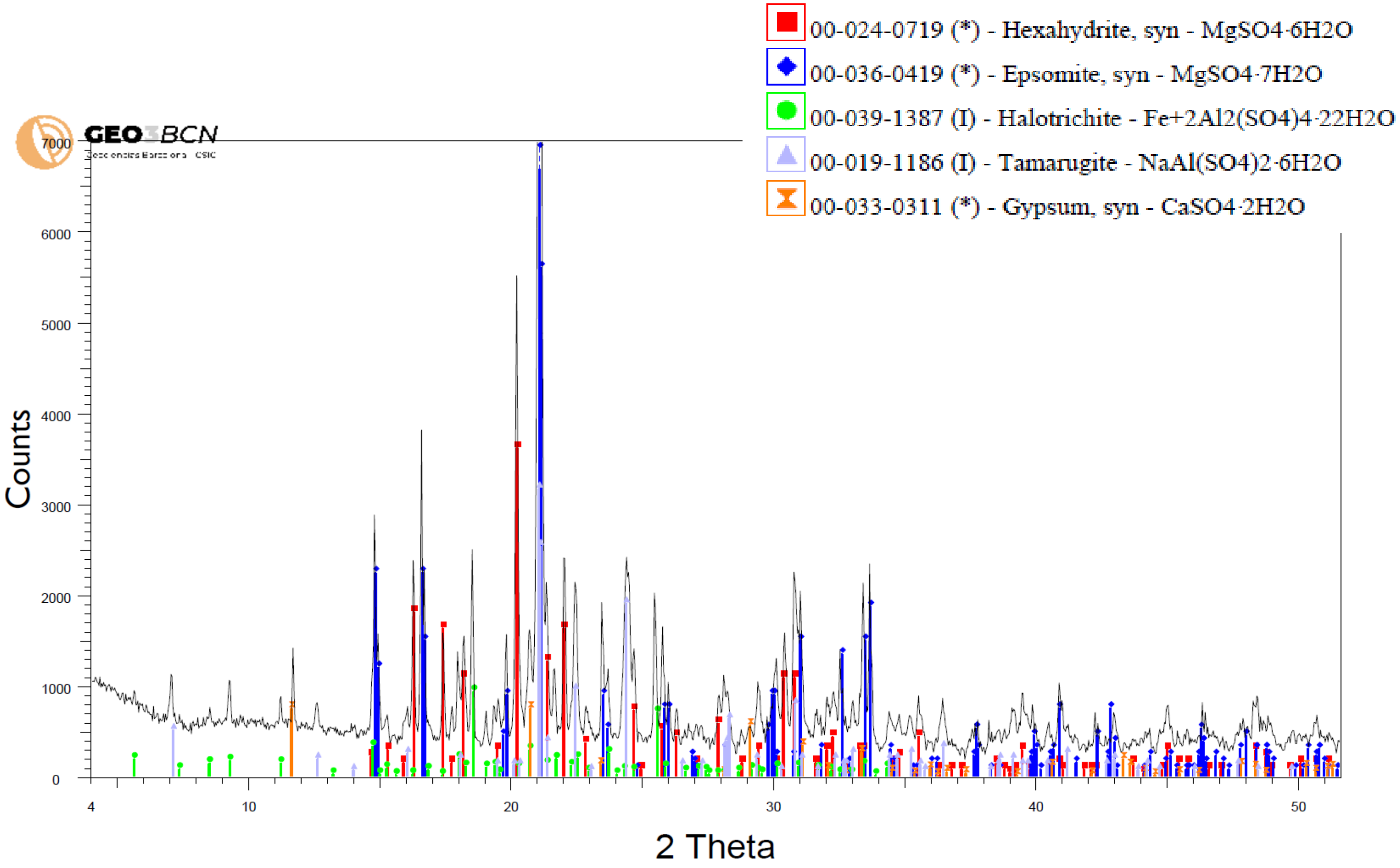

Figure S4: Powder x-ray diffraction scan of sample Ref. LP_E_F_a2, collected in the sampling point number 1 (see Figures 2 and 3). The scan is dominated by peaks of epsomite and hexahydrite. In addition, several other fumarolic mineral phases like halotrichite, tamarugite and gypsum are identified.

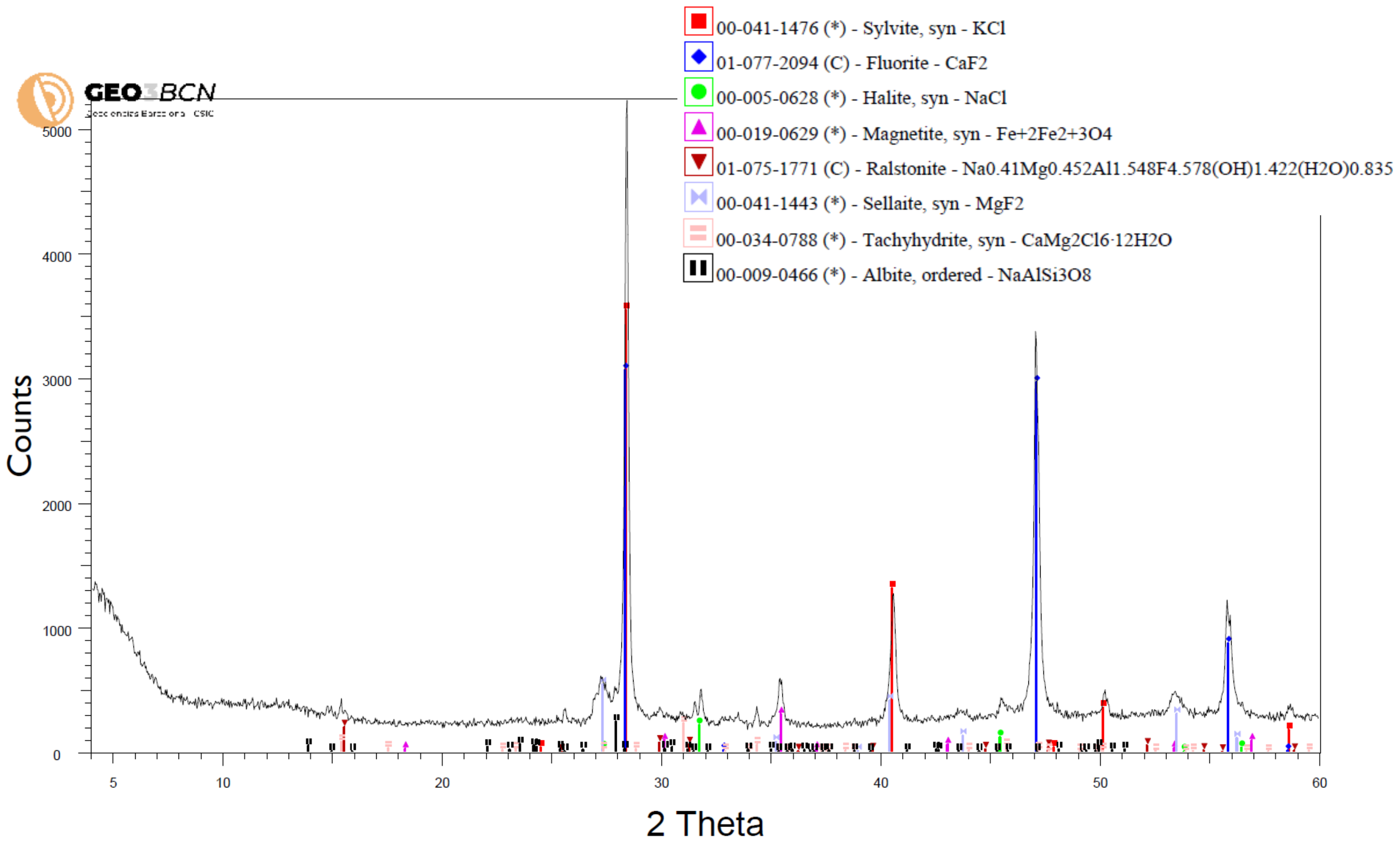

Figure S5: Powder x-ray diffraction scan of sample Ref. LP_E_F_k2, collected in the sampling point number 12 (see Figures 2 and 3). The scan is clearly dominated by the peaks of fluorite and sylvite. Sellaite and halite can also be identified from this scan. Other phases exhibit weaker peaks and can be tentatively attributed to different fumarolic and igneous minerals or their alteration products.

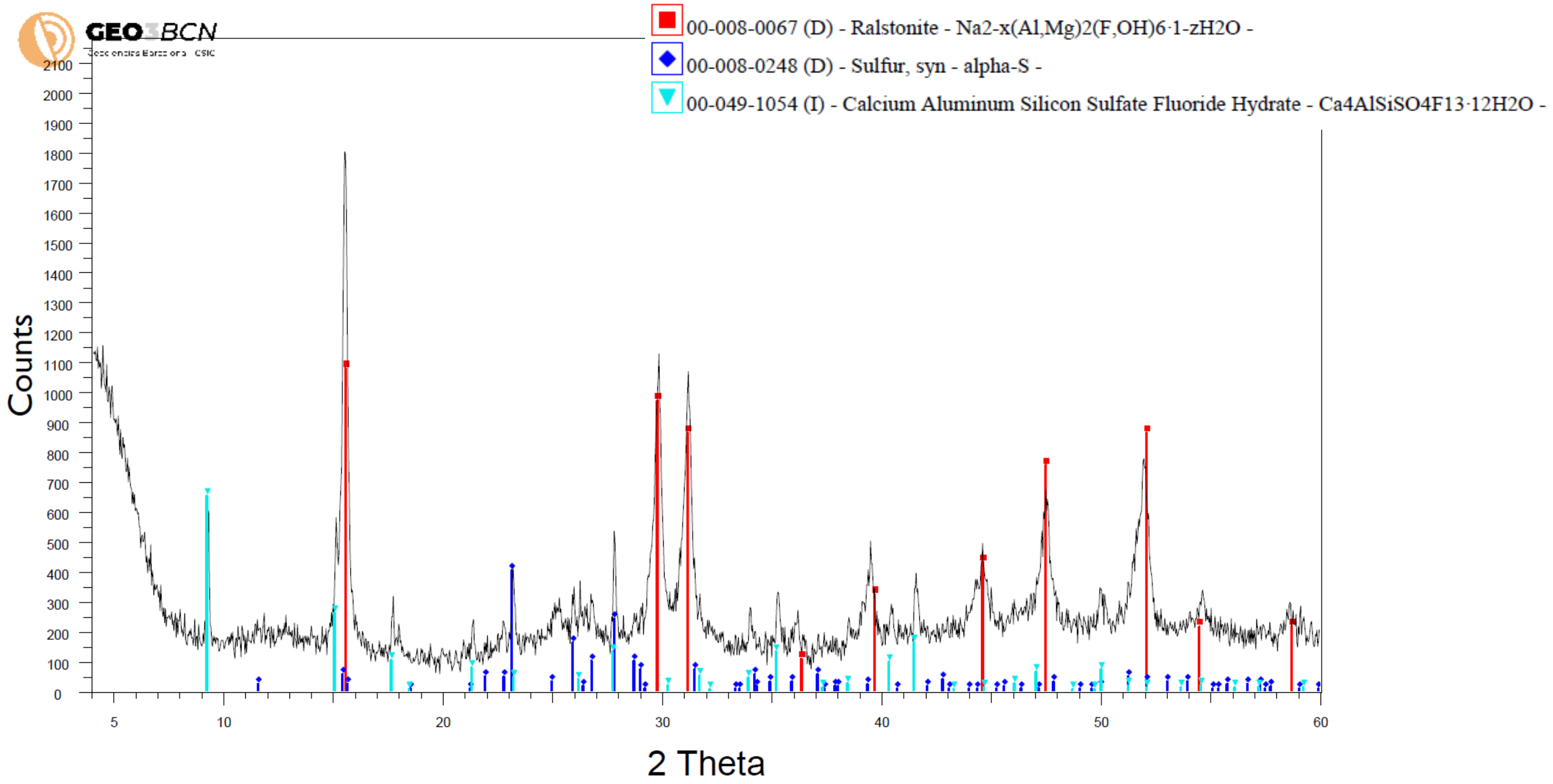

Figure S6: Powder x-ray diffraction scan of sample Ref. LP_E_F_m, collected in the sampling point number 14 (see Figures 2 and 3). The scan allows us to identify the fluoride mineral hydrokenoralstonite as well as meniaylovite. The latter is not included in the PDF-2 database in mineral form, but appears as a synthetic compound. The scan of this interesting sample also allows us to observe the occurrence of smaller amounts of native sulphur.

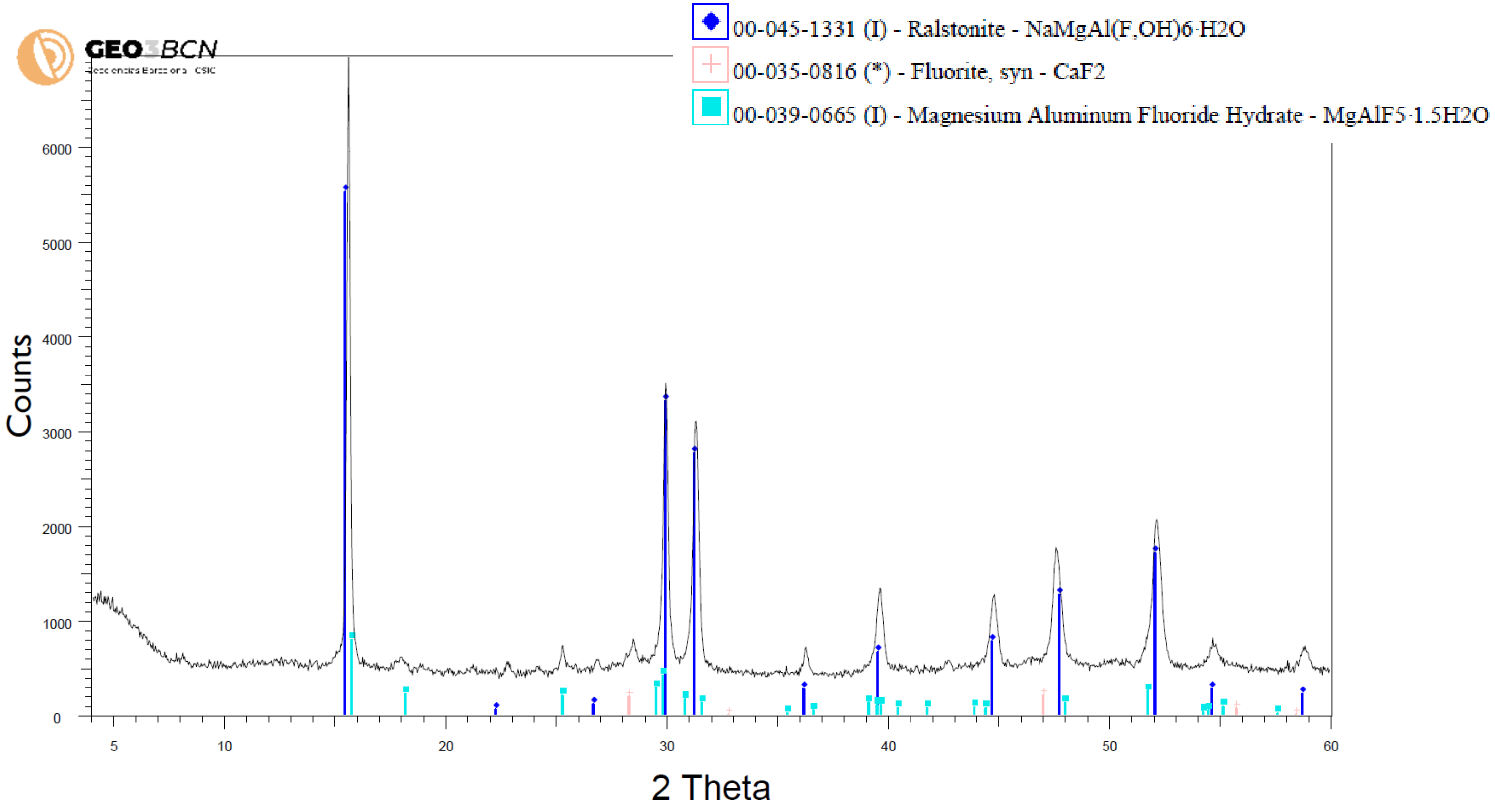

Figure S7: Powder x-ray diffraction scan of sample Ref. LP_E_F_n, collected in the sampling point number 15 (see Figures 2 and 3). The scan is clearly dominated by the reflections of the fluoride mineral hydrokenoralstonite. Very weak peaks from fluorite and leonardsenite (the magnesium aluminium fluoride hydrate phase) also occur in this scan.

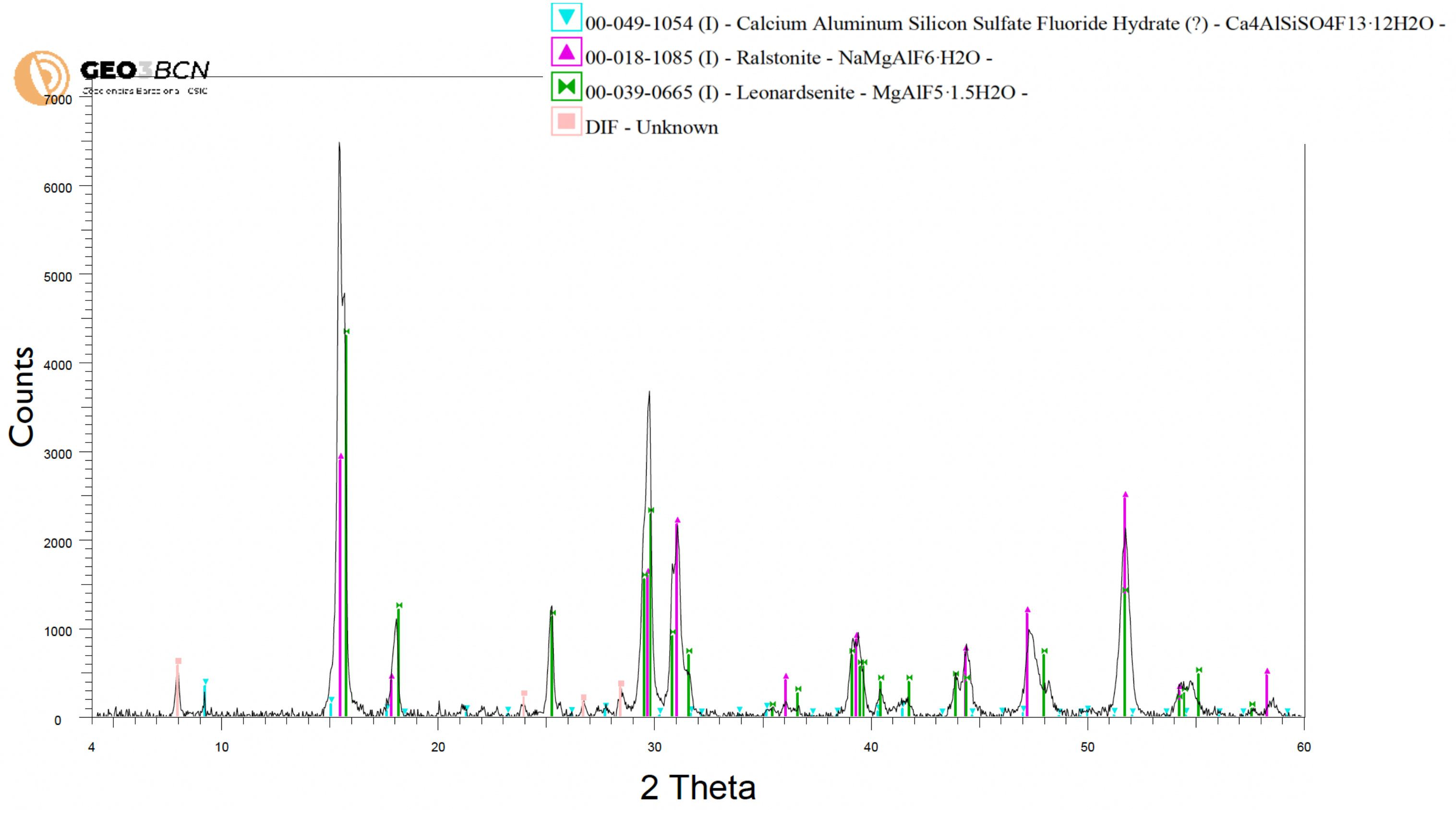

Figure S8: Powder x-ray diffraction scan of sample Ref. LP_E_F_n4, collected in the sampling point number 15 (see Figures 2 and 3). The scan is clearly dominated by the reflections of leonardsenite and hydrokenoralstonite. Very weak peaks of other fumarolic minerals like meniaylovite and an unidentified phase are also detected.

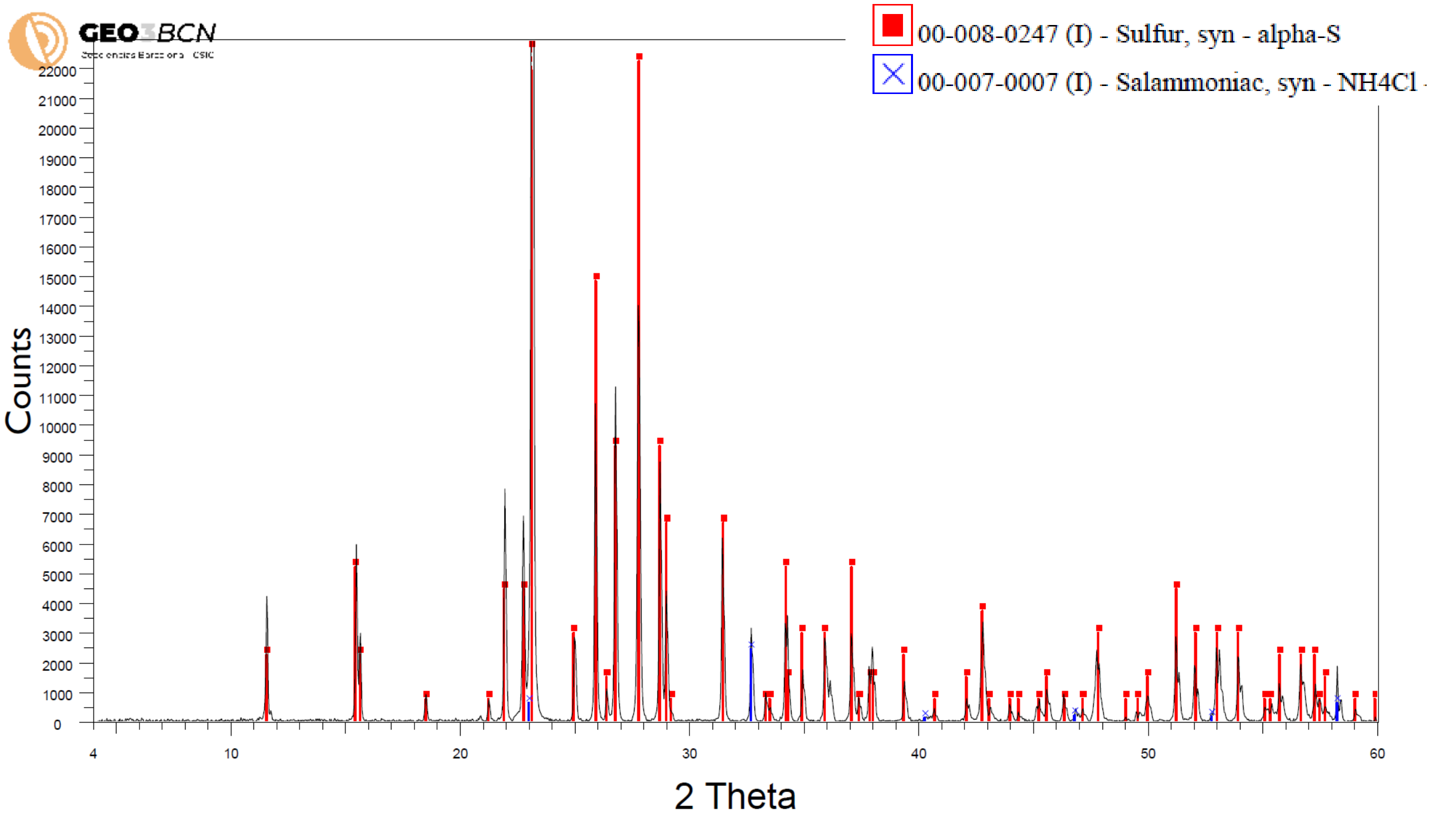

Figure S9: Powder x-ray diffraction scan of sample Ref. LP_E_J_06, collected in the sampling point number 21 (see Figures 2 and 3). This scan is dominated by strong reflections of elemental sulphur and allows identifying the presence of salammoniac.

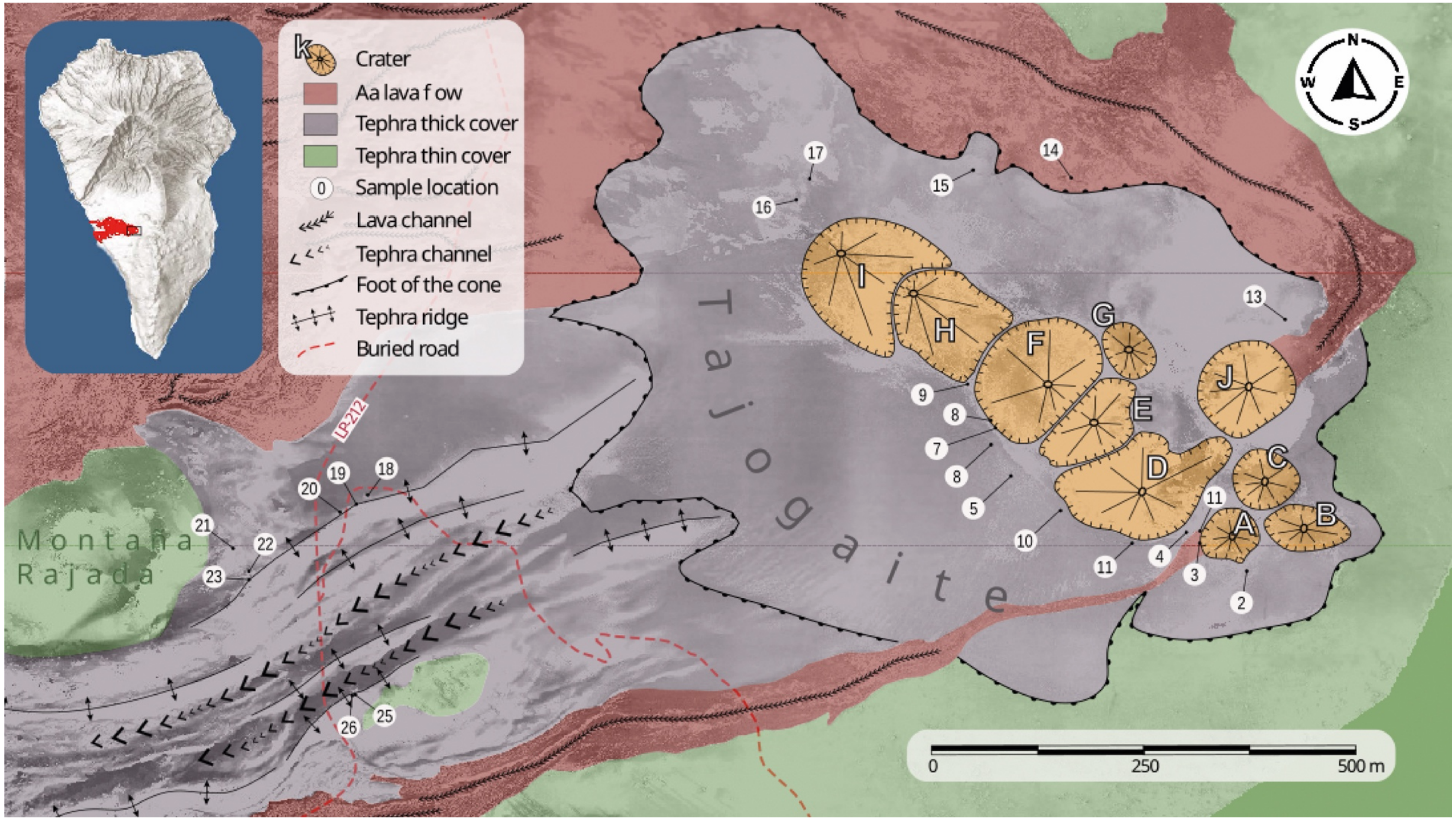

Figure S10: Tajogaite volcano geological map. "Tephra thin cover" refers to areas where lapilli and ash that do not completely cover pine trees (*Pinus canariensis*; ~5 metres). "Tephra thick cover" refers to areas where pine trees are completely buried. Individual craters are labelled by letters (A-J) and samples by numbers (1-32). Terrain relief information obtained from Cívico et al., 2022[29].

Locality	Country	native elements	sulphides and sulphosalts	chlorides	fluorides	carbonates	borates	sulphates	*Reference*
**Tajogaite**	La Palma, Spain	sulphur	-	erythrosiderite, halite, salammoniac, sylvite, tachyhydrite	fluorite, fluornatrocoulsellite, hydrokenoralstonite, leonardsenite, meniaylovite, sbachiite, sellaite, verneite	thermonatrite, trona	-	anhydrite, bassanite, blödite, burkeite, epsomite, gypsum, halotrichite, hexahydrite, jarosite, mascagnite, pickeringite, tamarugite	*Present study*
**Eldfell**	Iceland	sulphur	-	halite, salammoniac	cryptohalite, fluorite, hydrokenoralstonite, jakobssonite, leonardsenite, malladrite, meniaylovite, oskarssonite, sellaite	-	-	anhydrite, bassanite, eldfellite, gypsum, jarosite, langbeinite, tamarugite, thenardite	*Balić-Žunić et al., 2016*
**Hekla**		sulphur	-	halite, salammoniac	cryptohalite, fluorite, heklaite, hydrokenoralstonite, jakobssonite, leonardsenite, oskarssonite, pachnolite, rosenbergite, sbacchiite, verneite	-	-	anhydrite, glauberite, gypsum, thenardite	
**Surtsey**		sulphur	-	carnallite, halite	fluorite, meniaylovite, hydrokenoralstonite, sellaite	calcite, hydromagnesite	-	anhydrite, bassanite, glauberite, gypsum, kieserite, mirabilite, thenardite	
**Campi Flegrei**	Italy	sulphur	pararealgar, realgar	salammoniac	-	calcite	-	anhydrite, halotrichite, mascagnite, metavoltine, tschemirgite, voltaite	
**Etna**		sulphur	-	halite, kremersite, salammoniac, sylvite	fluorite, sellaite	calcite, trona	ameghinite	anhydrite, celestine, cossaite, halotrichite, mirabilite, thenardite	
**Vesubio**		sulphur, tellurium	covellite, chalcopyrite, galena, millerite, pararealgar, pyrite, pyrrhotite, pyrite, realgar	avogradrite, cottunite, erythrosiderite, halite, kremersite, molysite, salammoniac, sylvite	avogadrite, atacamite, cryptohalite, ferruccite, fluorite, parascandolaite, sellaite	thermonatrite, trona	sassolite	anhydrite, bassanite, jarosite, mascagnite, mirabilite, palmierite, voltaite	
**Fossa Crater (Vulcano)**		sulphur, tellurium	bismuthinite, cannizzarite, cosalite, demicheleite, galenobismutite, lillianite, pyrite, pyrrhotite, realgar, sphalerite, wurtzite	argesite, kremersite, panichiite, salammoniac, steropesite	demartinite, gearksutite, knasibfite, malladrite	-	clinometaborite, metaborite, sassolite	alunite, anhydrite, mascagnite, metavoltine	
**Fogo Volcano**	Cape Verde	sulphur	-	halite	hydrokenoralstonite	-	-	anhydrite, bassanite, biancite, blödite, gypsum, picromerite, tamarugite, thenardite	*Silva et al., 2019*
**Salton Sea**	California, USA	sulphur	-	bischofite, halite, salammoniac	-	-	-	anhydrite, bassanite, blödite, boussingaultite, epsomite, glauberite, gypsum, halotrichite, hexahydrite, jarosite, koktaite, konyaite, lecontite, mascagnite, mirabilite, pickeringite, tamarugite, thenardite, tschemirgite	*Adams et al., 2017*
**Sunset Crater**	Arizona, USA	sulphur	-	halite	fluorite, hydrokenoralstonite	calcite	-	alunite, baryte, celestine, gypsum, hexahydrite, jarosite, pickeringite, voltaite	*Hanson et al., 2010*
**Mutnovsky**	Kamchatka, Russia	sulphur	-	salammoniac	-	-	-	alunite, amarillite, baryte, copiapite, gypsum, halotrichite, jarosite, lonecreekite, letovicite, mascagnite, melanterite	Zhitova et al., 2022

Table S1: Mineralogy of different fumarolic localities worldwide. Comparison with the Tajogaite volcano fumaroles (present study). Data for comparison obtained from Balić-Žunić et al., 2016[34]; Silva et al., 2019[52]; Adams et al., 2017[2]; Hanson et al., 2010[57] and Zhitova et al., 2022[58].
